# mTOR inhibition prevents angiotensin II–induced aortic rupture and pseudoaneurysm but promotes dissection in *Apoe*-deficient mice

**DOI:** 10.1172/jci.insight.155815

**Published:** 2022-02-08

**Authors:** Changshun He, Bo Jiang, Mo Wang, Pengwei Ren, Sae-Il Murtada, Alexander W. Caulk, Guangxin Li, Lingfeng Qin, Roland Assi, Constantinos J. Lovoulos, Martin A. Schwartz, Jay D. Humphrey, George Tellides

**Affiliations:** 1 Department of Surgery (Cardiac), Yale School of Medicine, New Haven, Connecticut, USA.; 2 Department of Biomedical Engineering, Yale School of Engineering and Applied Science, New Haven, Connecticut, USA.; 3 Vascular Biology and Therapeutics Program, Yale School of Medicine, New Haven, Connecticut, USA.; 4 Veterans Affairs Connecticut Healthcare System, West Haven, Connecticut, USA.; 5 Department of Surgery, Frank H. Netter MD School of Medicine, Quinnipiac University, North Haven, Connecticut, USA.; 6 Department of Medicine (Cardiology),; 7 Department of Cell Biology, and; 8 Yale Cardiovascular Research Center, Yale School of Medicine, New Haven, Connecticut, USA.

**Keywords:** Vascular Biology, Cardiovascular disease, Mouse models, Surgery

## Abstract

Aortic dissection and rupture are triggered by decreased vascular wall strength and/or increased mechanical loads. We investigated the role of mTOR signaling in aortopathy using a well-described model of angiotensin II–induced dissection, aneurysm, or rupture of the suprarenal abdominal aorta in *Apoe*-deficient mice. Although not widely appreciated, nonlethal hemorrhagic lesions present as pseudoaneurysms without significant dissection in this model. Angiotensin II–induced aortic tears result in free rupture, contained rupture with subadventitial hematoma (forming pseudoaneurysms), dilatation, or healing, while the media invariably thickens regardless of mural tears. Medial thickening results from smooth muscle cell hypertrophy and extracellular matrix accumulation, including matricellular proteins. Angiotensin II activates mTOR signaling in vascular wall cells, and inhibition of mTOR signaling by rapamycin prevents aortic rupture but promotes dissection. Decreased aortic rupture correlates with decreased inflammation and metalloproteinase expression, whereas extensive dissection correlates with induction of matricellular proteins that modulate adhesion of vascular cells. Thus, mTOR activation in vascular wall cells determines whether aortic tears progress to dissection or rupture. Previous mechanistic studies of aortic aneurysm and dissection by angiotensin II in *Apoe*-deficient mice should be reinterpreted as clinically relevant to pseudoaneurysms, and mTOR inhibition for aortic disease should be explored with caution.

## Graphical abstract

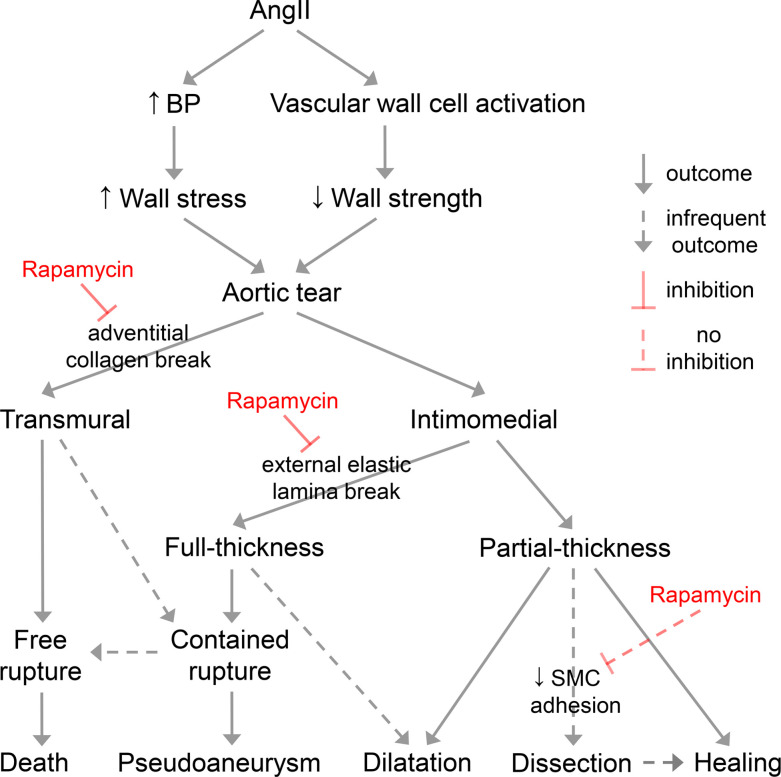

## Introduction

The aorta serves as the primary conduit for conveying blood from the heart to the body, and its wall consists of 3 functionally distinct layers, the intima, media, and adventitia. Its wall becomes compromised structurally when mechanical stress exceeds strength, leading to a constellation of life-threatening conditions termed acute aortic syndrome (1). Dissection is defined as a tear in the aortic wall that allows blood to separate layers of the media. Classic dissection, the most common manifestation, exhibits an intimal entrance site with blood present within a false lumen in the media, whereas intramural hematoma presents with thrombus in the media without intimal tears, and penetrating atherosclerotic ulcers can bleed into the media. In contrast, rupture is a tear through the aortic wall, resulting in extramedial hemorrhage that is either contained by the adventitia and/or surrounding tissues or extravasated with free accumulation within body cavities. Overlap of these complications is possible as dissections can perforate through the vascular wall and rupture sites may have associated medial ecchymosis. Moreover, intimal tears extending into the media but without blood between medial lamellae give rise to aortic wall defects considered a variant form of dissection that contribute to aneurysm formation (2). These lesions, termed incomplete dissection or limited intimal tears, may expose the adventitia if the media is completely disrupted (3). Notably, the initial description of limited dissection considered the lesion along a spectrum of mural lacerations involving varying depths of the vascular wall, ranging from classic dissection to partial and transmural rupture (4).

Although dissection and rupture can occur in nondilated aortas, they are more frequent in dilated vessels. Additionally, a dissected nondilated aorta can enlarge over time to become aneurysmal. An aneurysm is defined as a localized dilatation of an artery with luminal enlargement greater than 50% of expected diameter (5). A pseudoaneurysm results when a contained tear of the vascular wall gives the external appearance of an enlarged aorta but is due to accumulated blood within a false lumen lined by fibrous tissue, unlike the wall of true aneurysms consisting of all 3 layers, though remodeled (6). Pseudoaneurysms may occur spontaneously in diseased aortas but usually result from trauma and iatrogenic complications (7). Management of acute aortic syndrome depends on the location of the lesion. Dissection and rupture of the proximal aorta require urgent surgical treatment because mortality is high. In contrast, dissection of the descending thoracic and abdominal aorta may be managed medically depending on complications, although contained and free rupture of the aorta necessitate intervention regardless of the segment (8).

Experimental models in animals, particularly rodents, have provided great insight into mechanisms of aortic disease. A common model uses a continuous high rate of infusion of angiotensin II (AngII) in apolipoprotein E–null (*Apoe*^–/–^) mice, first described over 20 years ago as a model of abdominal aortic aneurysm (9). Subsequent analyses termed the initial lesion as aortic dissection leading to aneurysmal dilatation (10, 11). Regardless, continuous AngII infusion has been used in mice harboring diverse genetic mutations to study pathological mechanisms as well as to elucidate efficacy of candidate pharmacological interventions (12–15). We adopted this model to investigate the role of mTOR signaling in aortic disease. As previously reported, AngII infusion in *Apoe*^–/–^ mice often results in hematomas of the suprarenal abdominal aorta or sudden deaths from aortic rupture. We interpret these hemorrhagic lesions as full-thickness intimomedial tears with pseudoaneurysm, rather than as aneurysms or dissections. Importantly, we found that AngII infusion activated mTOR signaling in smooth muscle cells (SMCs) of the media, and mTOR inhibition by rapamycin prevented aortic rupture and pseudoaneurysm. Nonetheless, rapamycin also enabled extensive de novo dissection associated with persistent induction of several nonstructural extracellular matrix (ECM) proteins having counteradhesive functions in vascular cells.

## Results

### AngII induces aortic rupture and pseudoaneurysm.

**Figure 1:**
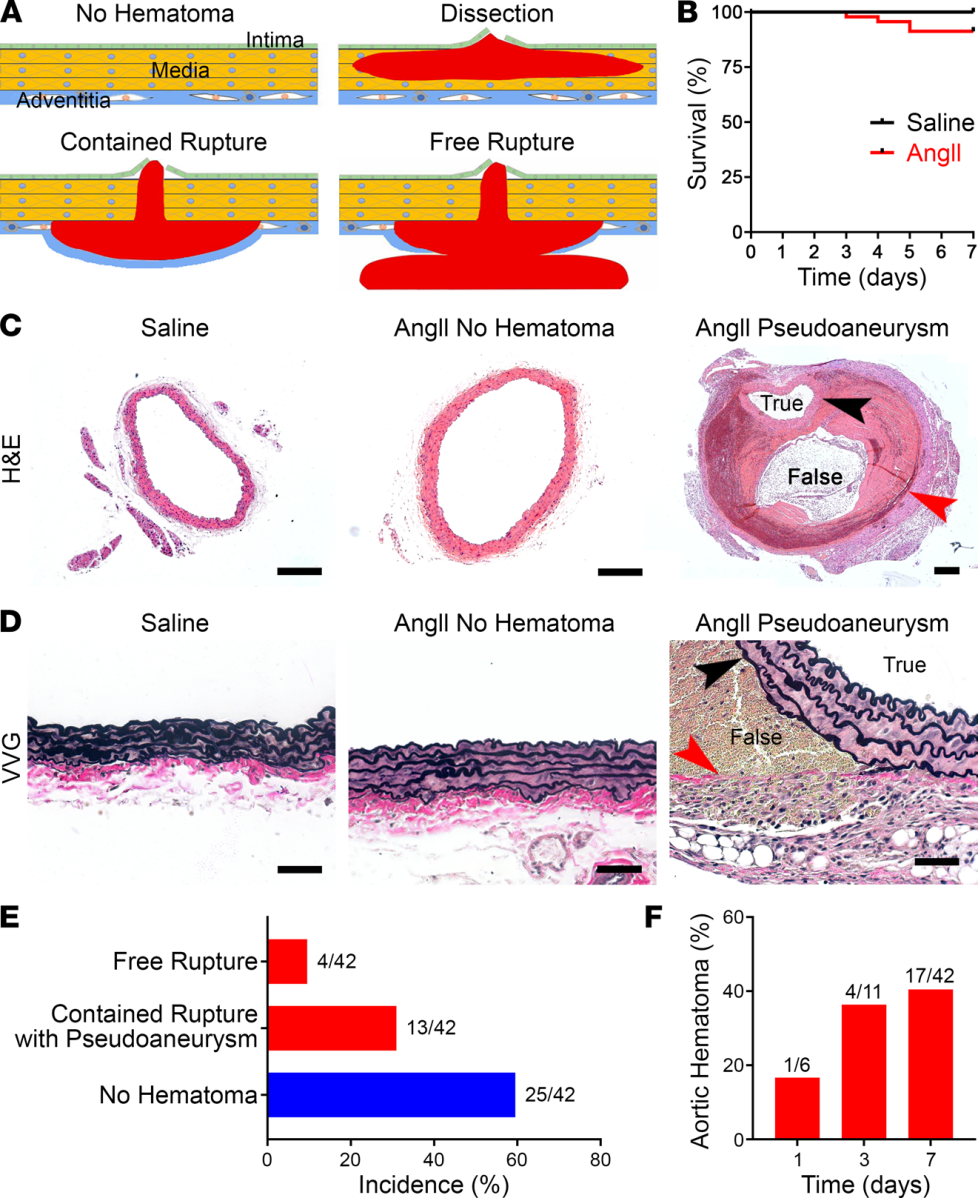
AngII induces aortic rupture and pseudoaneurysm. (**A**) The aortic wall consists of intimal, medial, and adventitial layers. A tear of the inner wall can lead to dissection (accumulation of blood between elastic laminae). Alternatively, a tear through the aortic wall can lead to either contained rupture (hemorrhage contained by the adventitia or periaortic tissue forming a pseudoaneurysm as it enlarges) or free rupture (extravasation into body cavities, often with exsanguination). (**B**) Survival of *Apoe*^–/–^ mice infused with saline (*n* = 11) or AngII (*n* = 42) for 7 days. (**C**) H&E stains of suprarenal abdominal aortas of saline- or AngII-infused *Apoe*^–/–^ mice without or with hemorrhagic lesions, scale bars: 200 μm. Note lesser magnification of right panel to include the large pseudoaneurysm containing blood and thrombus within a false lumen between the media (black arrowhead) and adventitia (red arrowhead). (**D**) Verhoeff-Van Gieson stains showing thrombus between the external elastic lamina (black arrowhead) and adventitial collagen fibers (red arrowhead) of a pseudoaneurysm; although the medial laminae widen in AngII-infused aortas, no erythrocytes are detected within. Scale bars: 50 μm. (**E**) Incidence of aortic complications after AngII treatment for 7 days. (**F**) Incidence of AngII-induced aortic hematomas at 1, 3, and 7 days.

Surviving animals were euthanized after 7 days of AngII-infusion, and the suprarenal abdominal aorta was examined using a dissecting microscope after saline flush of luminal blood. Standard histology further allowed hemorrhagic lesions, if present, to be classified as dissection, namely, blood within the media and/or rupture, that is, accumulation of blood outside of the media, either contained or free (Figure 1A). The few animals (~10%) that died prematurely at 3 to 5 days exhibited free rupture of the aorta, with blood found in the peritoneal cavity at necropsy (Figure 1B). A minority of animals (~30%) that survived to 7 days had grossly visible hematomas of the suprarenal abdominal aorta. Histomicroscopy revealed pseudoaneurysms in all of these hemorrhagic lesions, that is, large contained hematomas of the aorta with blood separating the media from the adventitia to form a false lumen distinct from the true lumen; erythrocytes were not visible within the elastic lamellae of the media (Figure 1, C–E). Some of these animals had associated hemorrhagic lesions of the thoracic aorta, although these proximal lesions were not studied further. The remaining animals (~60%) had no gross aortic pathology at 7 days. In additional animals examined before 7 days, aortic hematomas were found as early as 1 day and reached near peak incidence by 3 days (Figure 1F). Thus, AngII rapidly induced either lethal free rupture or contained rupture with pseudoaneurysm of the suprarenal abdominal aorta within 1 week in approximately half of the *Apoe*^–/–^ mice.

### AngII-induced aortic tears do not cause significant dissection.

**Figure 2:**
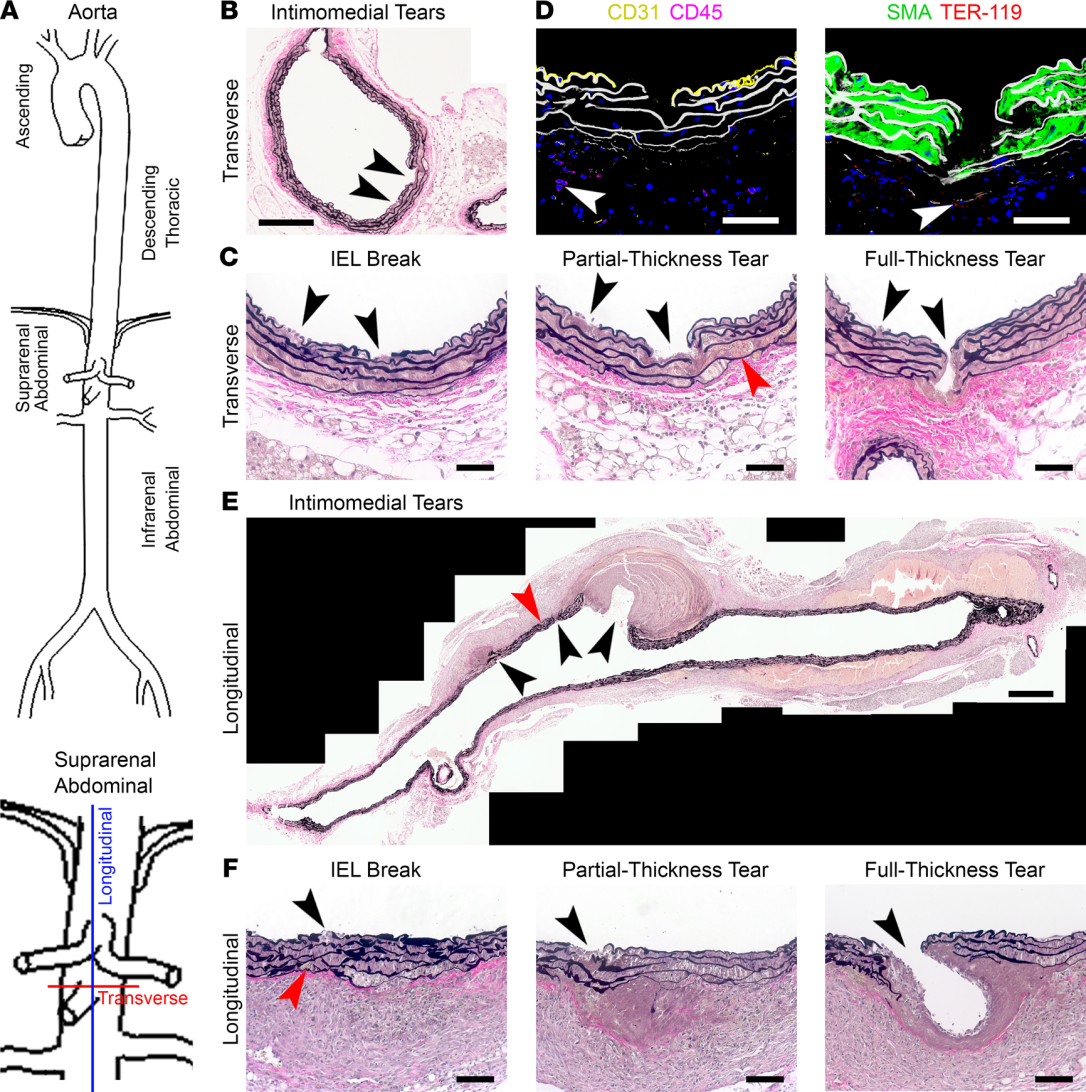
Spectrum of AngII-induced aortic tears. *Apoe*^–/–^ mice were infused with AngII for 7 days. (**A**) Suprarenal abdominal aortic segments were sectioned transversely or longitudinally and analyzed by Verhoeff-Van Gieson stain or immunostains. (**B**) Transverse section without visible hematoma showing 2 intimomedial tears (black arrowheads). (**C**) Higher magnification serial sections showing progression of the intimomedial tears (black arrowheads) of **A**, 1 of which (left) remains a superficial lesion with a limited break of the internal elastic lamina (IEL) and the other (right) extends farther into the media as a partial-thickness tear, with breaks of several elastic laminae and limited erythrocytes between the outer laminae (red arrowhead), and then as a full-thickness tear breaking the external elastic lamina. (**D**) Confocal images of intimomedial tears delineating CD31^+^ endothelial cells (yellow, left panel), SMA^+^ SMCs (green, right panel), and elastic laminae (white) with extravasation of TER-119^+^ erythrocytes (red, right panel, white arrowhead), infiltration of CD45^+^ leukocytes (purple, left panel, white arrowhead), and DAPI-labeled nuclei (blue). (**E**) Longitudinal section with a visible hematoma showing 3 intimomedial tears (black arrowheads), 1 with a small contained rupture (left), another with no extramedial blood (center) but a nearby limited dissection of the media (red arrowhead), and a third with a contained rupture forming a large thrombus-filled pseudoaneurysm (right). (**F**) Higher magnification serial sections showing progression of the left intimomedial tear of **D** (black arrowheads), from a superficial lesion with a break of the internal elastic lamina and limited erythrocyte accumulation in the outer laminae (red arrowhead), extending into the media as a partial-thickness tear with breaks of several elastic laminae, to a full-thickness tear breaking the external elastic lamina. Composite photomicrographs (**A** and **D**), scale bars: 50 μm (**B**, **C**, and **E**), 200 μm (**A**), and 500 μm (**D**).

To determine how rupture developed in response to AngII infusion, we further examined lesions in select specimens where serial histological sections avoided misidentification of artifacts resulting from cutting or associated with branch origins (Figure 2A). Intimal and medial tears of varying depths were found near branch orifices even in aortas without visible hematomas, ranging from breaks of the internal elastic lamina with no evident sequelae to full-thickness intimomedial separation without extramedial blood or small contained ruptures without or with limited dissection (Supplemental Figure 1, A–C; supplemental material available online with this article; https://doi.org/10.1172/jci.insight.155815DS1). Serial sections of one aorta without pseudoaneurysm revealed progressive abnormalities: a) minimal intimomedial tear with a break in the internal elastic lamina, b) partial-thickness intimomedial tear with breaks in several elastic laminae but sparing the external elastic lamina, and c) full-thickness intimomedial tear crossing all elastic laminae and forming a small contained rupture (Figure 2, B and C). Confocal microscopy confirmed that CD31^+^ endothelial cells, smooth muscle α-actin–positive (SMA^+^) SMCs, and elastic laminae were crossed by intimomedial tears along a radial plane with subadventitial presence of TER-119^+^ erythrocytes and adventitial infiltration of CD45^+^ leukocytes (Figure 2D). Intimomedial defects of varying depth were also found in aortas with gross hematomas (Supplemental Figure 1, D–F). Longitudinal sections were examined in 3 specimens to increase the yield of associated lesions, although this tissue orientation had a greater susceptibility to cutting artifacts. One pseudoaneurysm had multiple intimomedial tears with differing manifestations, ranging from fibrous healing to small and large contained ruptures (Figure 2E). Serial sections documented a progression of lesions to full-thickness intimomedial tears (Figure 2F and Supplemental Figure 1G). The pseudoaneurysm was associated with minimal dissection of the media, namely less than 200 μm length (or <1% of the visualized aortic wall length). In 2 other pseudoaneurysms sectioned longitudinally, erythrocytes between elastic lamellae were either absent or more limited in extent (Supplemental Figure 2). Thus, AngII-induced aortic tears were associated with various outcomes, including superficial ulcer, thrombus, tissue repair, limited dissection, contained rupture, pseudoaneurysm, and free rupture, but not isolated or extensive dissection sufficient to cause macroscopic hematomas.

### AngII-induced aortopathy includes marked medial thickening.

**Figure 3:**
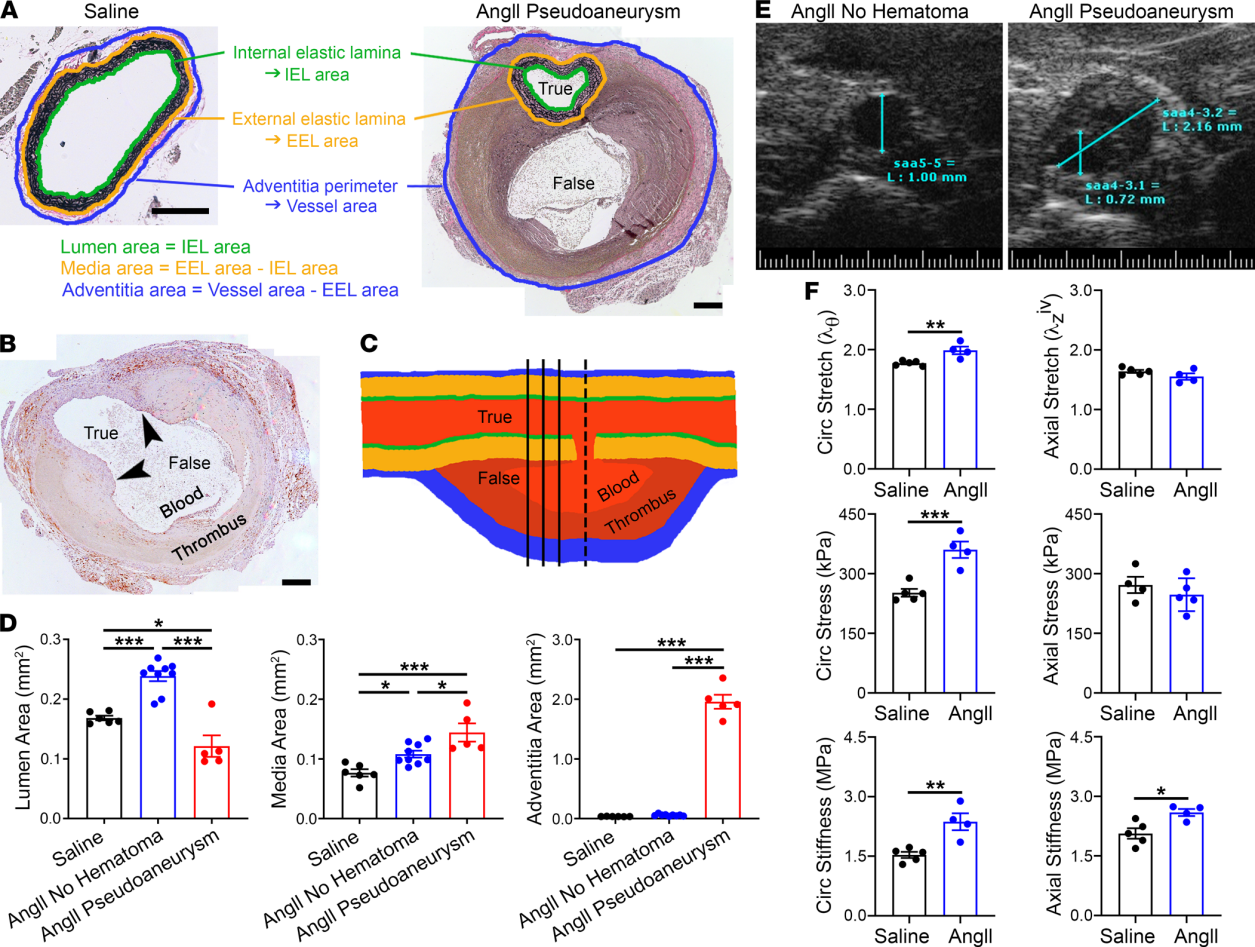
AngII-induced aortopathy is characterized by medial thickening and altered mechanics. (**A**) Aorta compartment areas were calculated from perimeter measurements of the internal and external elastic laminae and outer adventitia. Note lesser magnification of the pseudoaneurysm with a false lumen distinct from the true lumen and inclusion of the contained rupture within the adventitia area, scale bars: 200 μm. (**B**) Infrequent section (the example is of an F4/80 immunostain) showing a focal break of the intima and media (arrowheads) connecting the true to false lumen containing blood and thrombus, scale bar: 200 μm. Sections in which it was not possible to delineate uninterrupted IEL and EEL perimeters were not included for histomorphometry analysis. (**C**) Schematic of a pseudoaneurysm to illustrate more frequent transverse sections (solid lines) with the true lumen distinct from the false lumen versus an infrequent transverse section (interrupted line) where the true lumen opens into the false lumen. (**D**) Lumen, media, and adventitia areas of suprarenal abdominal aortas in *Apoe*^–/–^ mice infused with saline (*n* = 6) or AngII for 7 days without (*n* = 9) or with (*n* = 5) pseudoaneurysms. (**E**) Ultrasound images of AngII-infused aortas with unobstructed lumen (diameter = 1 mm) or with pseudoaneurysm (diameter = 2.2 mm) and smaller true lumen (diameter = 0.7 mm). (**F**) Biomechanical testing of nonruptured suprarenal aortas from *Apoe*^–/–^ mice infused with saline (*n* = 5) or AngII (*n* = 4) for circumferential (circ) and axial stretch, stress, and stiffness. Higher circumferential stretch and stress at elevated blood pressure indicate a lack of effective tissue-level remodeling of structural proteins. Individual data shown, bars represent mean ± SEM. *P < 0.05, **P < 0.01, ***P < 0.001, unpaired, 2-tailed *t* test (**F**) and 1-way ANOVA with Tukey’s multiple-comparison test (**D**).

Morphometric analysis traced the perimeters of the internal and external elastic laminae and the outer adventitia to calculate enclosed cross-sectional areas of the primary vascular compartments (Figure 3A). The intima was of negligible thickness and was included with measurements of the true lumen. Focal breaks of the intima and media connecting the true and false lumens were rare, and these infrequent sections were not evident in elastin stains used for morphometric measurements (Figure 3, B and C). We separately analyzed AngII-treated aortas with or without hematomas. Luminal and medial areas increased moderately in the absence of hematomas; changes in the adventitial area did not reach statistical significance (Figure 3D). After aortic rupture and pseudoaneurysm formation, there was a reduction in cross-sectional area of the true lumen (likely secondary to compression by the pressurized false lumen), a greater increase of the media area, and marked enlargement of the cross section enclosed by the adventitia (attributable to the false lumen, enclosed thrombus, and adventitial thickening). Partial obstruction of the lumen secondary to pseudoaneurysm was confirmed in vivo by ultrasound (Figure 3E). AngII-induced wall remodeling (independent of hematoma) increased circumferential stretch and stiffness, yet thickening was insufficient to normalize wall stresses increased by the AngII-induced hypertension at 7 days (Figure 3F and Supplemental Table 1), consistent with previous analyses over 4 to 7 days of AngII infusion (16). Thus, AngII induced invariable medial thickening, variable changes in the lumen cross section ranging from dilatation without hematomas to encroachment with pseudoaneurysm, and significant enlargement of the area enclosed by the adventitia only after pseudoaneurysm formation.

### Medial thickening results from SMC hypertrophy and ECM accumulation.

**Figure 4:**
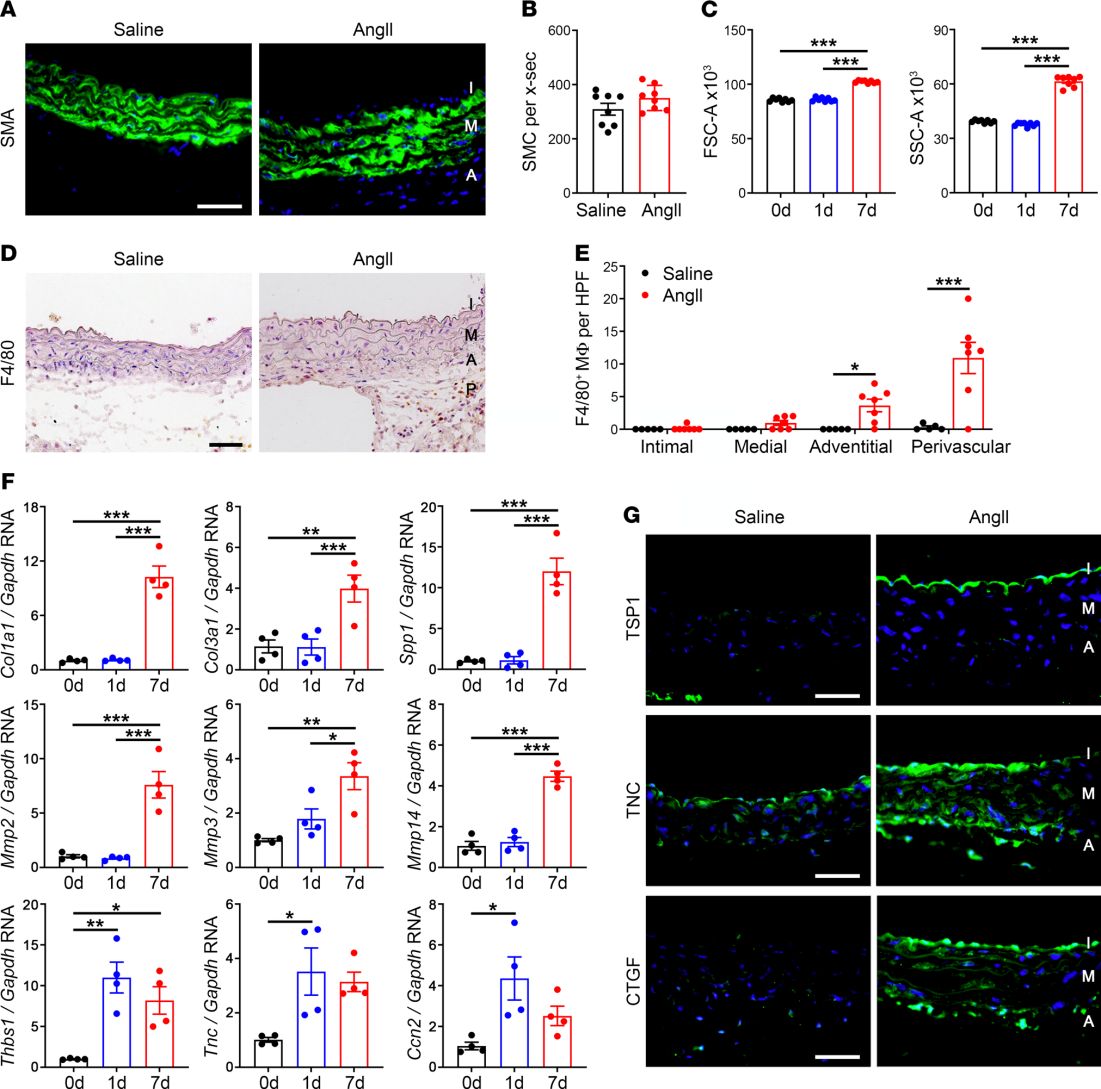
Medial thickening results from SMC hypertrophy and ECM accumulation. *Apoe*^–/–^ mice were infused with saline or AngII and the suprarenal abdominal aortas were analyzed after 0–7 days. (**A**) Immunofluorescence microscopy for SMA expression (green) with DAPI-labeled nuclei (blue) at day 7, scale bar: 50 μm. (**B**) Number of SMCs per cross section (x‑sec) extrapolated from counts of medial nuclei after 7 days (*n* = 8). (**C**) Flow cytometry for forward (FSC-A) and side (SSC‑A) scatter area of enzymatically isolated SMA^+^ SMCs at 0, 1, and 7 days (*n* = 8). (**D**) Immunostains for F4/80^+^ cells in intima (I), media (M), adventitia (A), and perivascular tissue (P) at 7 days, scale bar: 50 μm. (**E**) Number of F4/80^+^ macrophages (M) in vascular wall layers at 7 days (*n* = 5–7). (**F**) Quantitative RT-PCR for selected transcripts regulating ECM synthesis and degradation, namely *Col1a1*, *Col3a1*, *Spp1* (encoding osteopontin), *Mmp2*, *Mmp3*, *Mmp14*, *Thbs1*, *Tnc*, and *Ccn2* (encoding CTGF) at 0, 1, and 7 days (*n* = 4). (**G**) Expression of thrombospondin-1 (TSP1), tenascin-C (TNC), and CTGF (green) with DAPI-labeled nuclei (blue) at 7 days, scale bars: 50 μm. Individual data shown, bars represent mean ± SEM, *P < 0.05, **P < 0.01, ***P < 0.001, unpaired, 2-tailed *t* test (**B**), 1‑way ANOVA with Tukey’s multiple-comparison test (**C** and **F**), and 2-way ANOVA with Sidak’s multiple-comparison test (**E**).

To further characterize AngII-induced thickening of the media, we quantified changes in cellular and ECM components. After 7 days of AngII infusion, SMA^+^ SMCs appeared larger with greater separation between concentric cell layers (Figure 4A). The number of medial cells remained unchanged, however (Figure 4B). Flow cytometry of enzymatically isolated SMA^+^ SMCs revealed that AngII increased both forward scatter, indicative of cell size, and side scatter, indicative of cell granularity, at 7 days but not at 1 day of treatment (Figure 4C). There were few infiltrating macrophages within the thickened media of AngII-treated aortas despite significant accumulation in the adventitia and perivascular tissues (Figure 4, D and E). ECM synthesis and degradation were assessed by quantitative reverse transcription PCR (RT-PCR) in nonruptured aortas to minimize artifact from intramural blood. As expected in inflammatory and fibrotic conditions, 7-day infusions of AngII upregulated the expression of many ECM genes, including *Col1a1* (encoding collagen type 1, alpha 1), *Col3a1* (encoding collagen type III, alpha 1), and *Spp1* (encoding osteopontin), as well as matrix metalloproteinases (MMPs), including *Mmp2*, *Mmp3*, and *Mmp14* (Figure 4F). Interestingly, there was more rapid induction of several nonstructural ECM genes, namely *Thbs1* (encoding thrombospondin-1), *Tnc* (encoding tenascin-C), and *Ccn2* (encoding connective tissue growth factor, CTGF) within 1 day. Immunostaining demonstrated that thrombospondin-1 localized to the intima, while tenascin-C and CTGF were detected in all 3 layers of the vascular wall, including the media (Figure 4G). In summary, larger SMCs and accumulation of ECM contributed to the observed medial thickening.

### mTOR inhibition prevents aortic rupture and pseudoaneurysm but promotes dissection.

**Figure 5:**
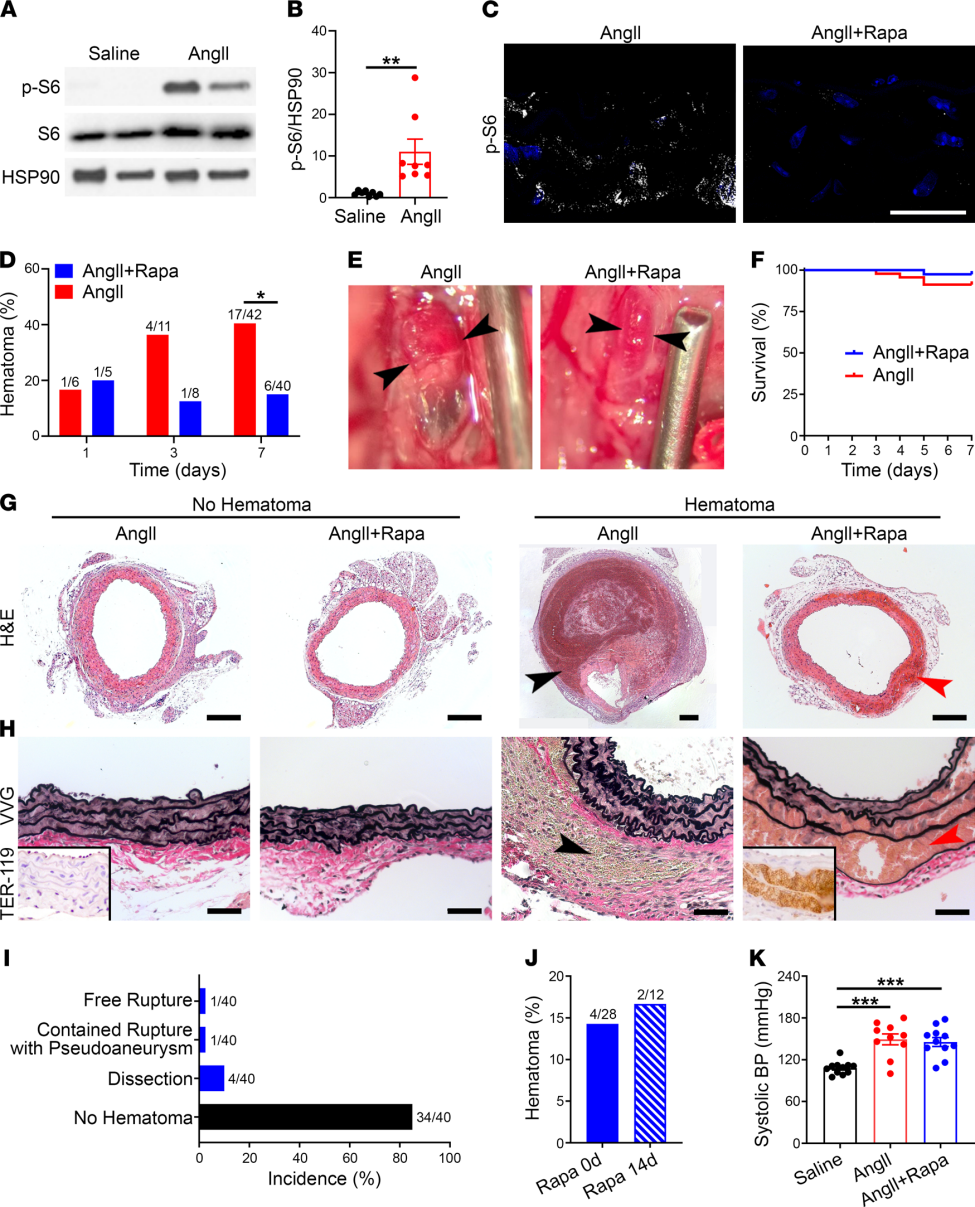
mTOR inhibition prevents aortic rupture and pseudoaneurysm but promotes dissection. *Apoe*^–/–^ mice were infused with AngII with or without rapamycin (Rapa) treatment for 7 days. (**A**) Western blot of aortas for phospho-S6 (p-S6) and S6. (**B**) Relative expression of phospho-S6 from pooled experiments (*n* = 8). (**C**) Confocal microscopy for phospho-S6 expression (white) and DAPI-labeled nuclei (blue) in media of suprarenal abdominal aortas, scale bar: 25 μm. (**D**) Incidence of aortic hematomas at 1–7 days. (**E**) Appearance of aortic hematomas (arrowheads), scale provided by needle of 1.27 mm outer diameter. (**F**) Survival of animals infused with AngII without (*n* = 42) or with (*n* = 40) rapamycin treatment for 7 days. (**G**) H&E stains showing medial thickening without hematomas compared with contained rupture (black arrowhead) and dissection (red arrowhead) in the absence or presence of rapamycin, respectively; scale bars: 200 μm (note different magnification of larger pseudoaneurysm). (**H**) Verhoeff-Van Gieson staining similarly shows thickened media without hematomas versus contained rupture (black arrowhead) and dissection (red arrowhead) in the absence or presence of rapamycin, respectively; TER-119 immunostain for erythrocytes in insets, scale bars: 50 μm. (**I**) Incidence of suprarenal abdominal aorta complications in AngII-infused *Apoe*^–/–^ mice treated with rapamycin. (**J**) Incidence of aortic hematomas in AngII-infused mice treated with rapamycin from day 0 to 7 or from day –14 to 7. (**K**) Systolic blood pressure (BP) in *Apoe*^–/–^ mice infused with saline (*n* = 11), AngII (*n* = 10), or AngII with rapamycin treatment (*n* = 11). Individual data shown for continuous variables, bars represent mean ± SEM, *P < 0.05, **P < 0.01, ***P < 0.001, unpaired, 2-tailed *t* test (**B**), Fisher’s exact test (**D** and **J**), and 1‑way ANOVA with Tukey’s multiple-comparison test (**K**).

To examine mechanisms for medial thickening, we considered mTOR signaling that is activated by various growth and stress stimuli in SMCs, including AngII (17). AngII infusion increased phosphorylation of S6, a sensitive readout of mTOR complex 1 signaling, within SMCs and other vascular wall cells of the suprarenal abdominal aorta (Figure 5, A–C). To determine whether mTOR signaling caused aortic rupture in this *Apoe*^–/–^ model, we treated the AngII-infused mice with the mTOR inhibitor rapamycin at 2 mg/kg/d every day (i.p.), a dose previously documented as effective in other murine models of aortopathy (18). Rapamycin therapy decreased mTOR signaling in SMCs and other vascular wall cells (Figure 5C and Supplemental Figure 3) and markedly diminished the incidence of aortic hematoma (a descriptive term that includes free ruptures) from 40% to 15% at day 7; the few remaining lesions appeared minimally enlarged and without darkly discolored mural thrombus (Figure 5, D and E). By survival and histological criteria, there were fewer aortic ruptures and pseudoaneurysms (Figure 5, F–I). Dissection of the media emerged, however, as delamination of elastic laminae with erythrocytes displacing SMCs; incidence of this isolated complication (10%) exceeded that of complete and incomplete ruptures combined (5%). Histological characteristics of intramural blood were confirmed by immunostaining for the erythroid lineage–specific antigen, TER-119. Initiating rapamycin administration 2 weeks prior to AngII infusion had similar outcomes as concomitant treatment (Figure 5J and Supplemental Figure 4). The modifying effects of rapamycin on AngII-induced aortopathy were independent of blood pressure (Figure 5K). Thus, mTOR inhibition greatly decreased the incidence of AngII-induced aortic rupture and pseudoaneurysm but spatially altered the site of mural hematomas, if present, from between the adventitial and medial layers to within the media.

### mTOR inhibition prevents inflammation and reduces MMPs but not medial thickening or induction of matricellular proteins.

**Figure 6:**
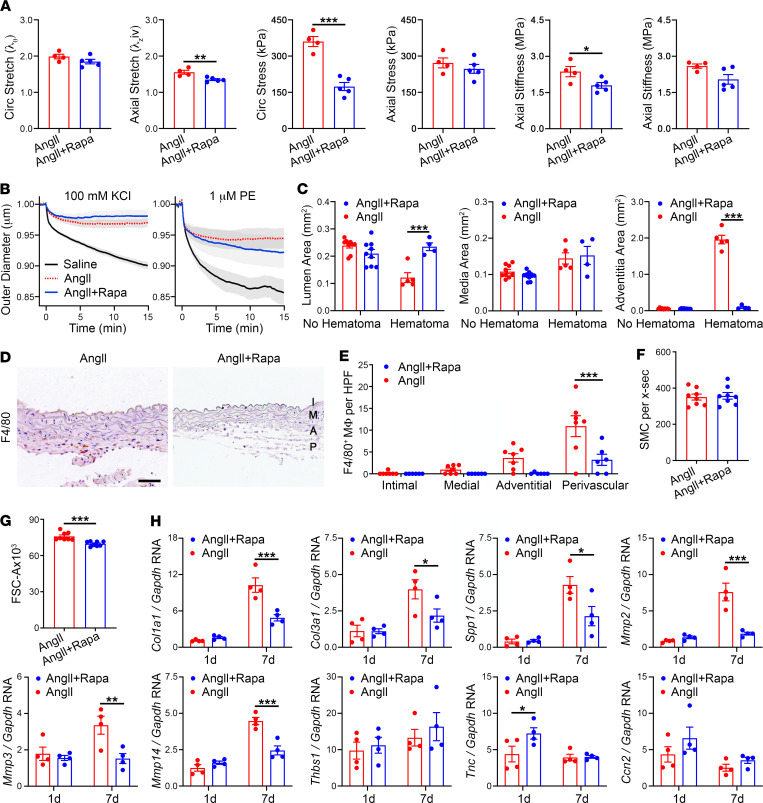
mTOR inhibition prevents inflammation and production of MMPs but not medial thickening or induction of matricellular proteins. *Apoe*^–/–^ mice were infused with AngII with or without rapamycin (Rapa) treatment, and the suprarenal abdominal aortas were analyzed at 7 days. (**A**) Biomechanical testing of AngII (*n* = 4) or AngII plus Rapa (*n* = 5) treated aortas for circumferential (circ) and axial stretch, stress, and material stiffness and (**B**) vasoconstriction responses to KCl and phenylephrine (PE) assessed by reduction of normalized outer diameter. (**C**) Lumen, media, and adventitia areas of suprarenal abdominal aortas without (*n* = 9) or with (*n* = 4–5) hematomas. (**D**) Immunostains for F4/80^+^ cells in intima (I), media (M), adventitia (A), and perivascular tissue (P) of aorta, scale bar: 50 μm. (**E**) Number of F4/80^+^ macrophages (M) per high power field (HPF) in vascular wall layers of aortas without hematomas (*n* = 6–7). (**F**) Number of SMCs per cross section (x-sec) of aortas without hematomas (*n* = 8). (**G**) Forward scatter area (FSC‑A) indicative of cell size of enzymatically isolated SMA^+^ SMCs (*n* = 8). (**H**) Quantitative RT-PCR for *Col1a1*, *Col3a1*, *Spp1*, *Mmp2*, *Mmp3*, *Mmp14*, *Thbs1*, *Tnc*, and *Ccn2* transcript levels at 1 or 7 days (*n* = 4). Individual data shown, bars represent mean ± SEM, *P < 0.05, **P < 0.01, ***P < 0.001, unpaired, 2-tailed *t* test (**A**, **F**, and **G**) and 2-way ANOVA with Sidak’s multiple-comparison test (**C**, **E**, and **H**) for AngII + Rapa versus AngII.

We further characterized how rapamycin modulates tissue and cellular responses in AngII-induced aortopathy. The relatively short duration of concomitant rapamycin therapy for 7 days did not reverse AngII-mediated SMC dysfunction (as indicated by altered potassium- and phenylephrine-induced vasoconstriction), but did improve circumferential stress and material stiffness, both highly mechano-regulated variables (Figure 6, A and B, and Supplemental Tables 1 and 2). Despite decreasing aortic complications, rapamycin did not prevent AngII-induced medial thickening in the absence or presence of hematoma (Figure 6C). Rapamycin did, however, protect against lumen loss and expansion of the cross section enclosed by the adventitia by averting pseudoaneurysm formation. mTOR inhibition also markedly decreased AngII-induced recruitment of F4/80^+^ macrophages and diminished SMC hypertrophy without changes in SMC numbers (Figure 6, D–G). Although rapamycin reduced the upregulation of transcripts for structural ECM proteins and MMPs at day 7 of AngII infusion, it did not prevent the early induction of transcripts encoding matricellular proteins, and RNA for tenascin-C increased (Figure 6H). Thus, recruitment of macrophages and production of MMPs that may degrade the ECM were associated with aortic rupture, whereas matricellular proteins known to regulate endothelial cell adhesion were associated with dissection.

### Rapamycin-insensitive CTGF inhibits SMC adhesion to exogenous ECM.

**Figure 7:**
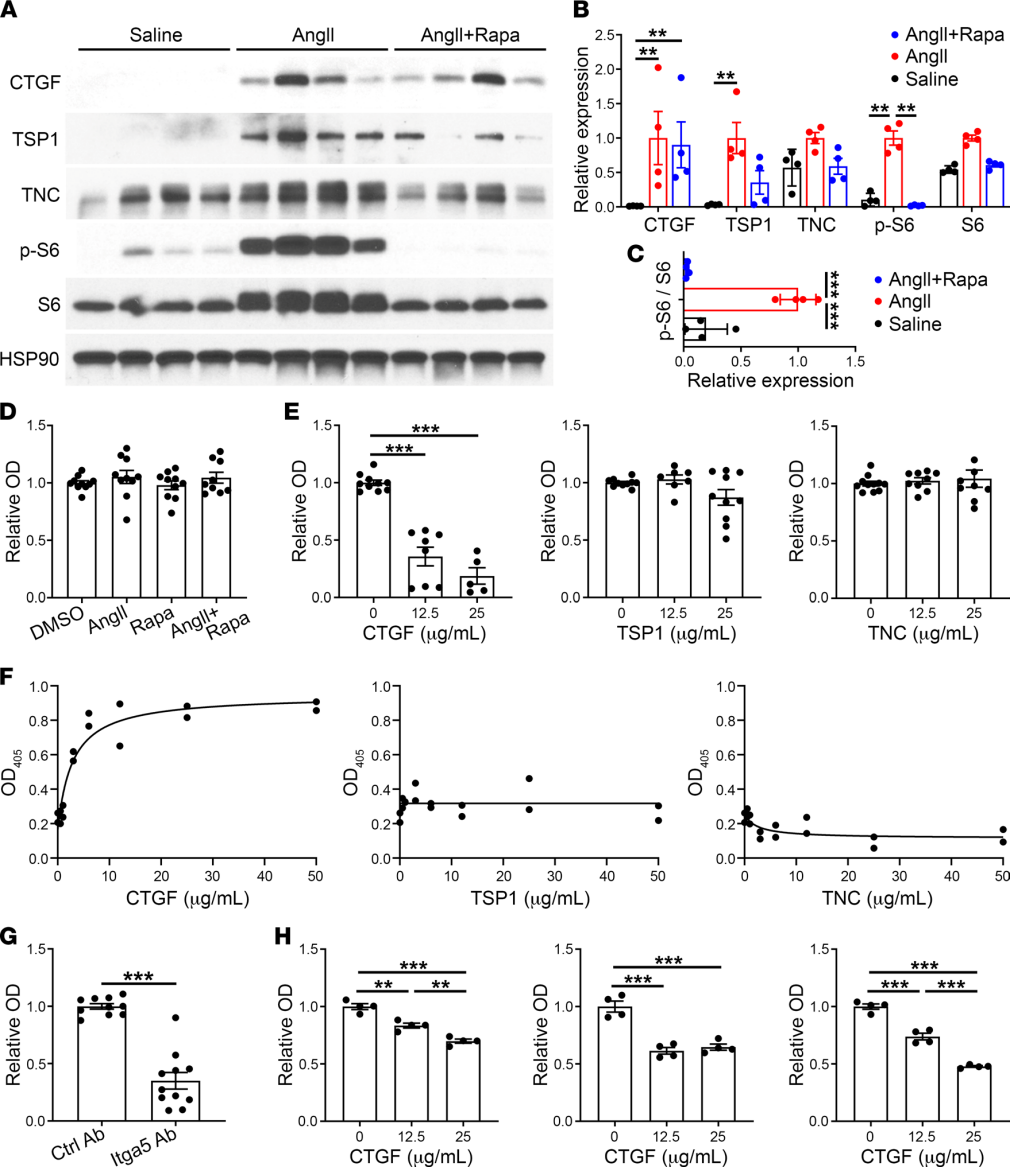
Rapamycin-insensitive CTGF inhibits SMC adhesion to exogenous ECM. (**A**) *Apoe*^–/–^ mice were infused with saline or AngII with or without rapamycin (Rapa) treatment for 7 days and the suprarenal abdominal aortas were analyzed by Western blot for CTGF, thrombospondin-1 (TSP1), tenascin-C (TNC), phospho-S6 (p-S6), and S6. (**B**) Densitometry of protein expression relative to HSP90 or (**C**) phospho-S6 expression relative to S6; expression normalized to peak levels with AngII treatment alone, (*n* = 4). (**D**) Colorimetric assay for number of murine aortic SMCs adherent to fibronectin-coated plates after 1 hour following cell pretreatment with vehicle, AngII at 100 nM, and/or rapamycin at 100 ng/mL for 45 minutes (*n* = 9–10, pooled from 3 experiments); OD_405_ readings normalized to vehicle-treated controls. (**E**) Similar fibronectin adhesion assay of SMCs pretreated with CTGF, thrombospondin-1, or tenascin-C at various doses for 45 minutes (*n* = 5–10, pooled from 3 experiments). (**F**) Colorimetric assay for number of SMCs adherent to plates coated with CTGF, thrombospondin-1, or tenascin-C at various concentrations (in the absence of fibronectin) after 1 hour (*n* = 2). (**G**) CTGF adhesion assay of SMCs pretreated with blocking antibody to integrin α_5_ (Itga5 Ab) or isotype-matched control antibody (Ctrl Ab) for 45 minutes (*n* = 10–11, pooled from 3 experiments). (**H**) Fibronectin adhesion assay of human aortic SMCs from 3 individuals pretreated with CTGF at various doses for 45 minutes (*n* = 4, shown separately for each subject). Individual data shown, bars represent mean ± SEM or lines represent nonlinear regression fitting by least-squares regression, **P < 0.01, ***P < 0.001, unpaired, 2-tailed *t* test (**G**), 1‑way ANOVA with Tukey’s multiple-comparison test (**C–E** and **H**), and 2‑way ANOVA with Sidak’s multiple-comparison test (**B**).

We verified that transcriptional expression of matricellular molecules corresponded with protein levels in vivo and examined the effects of matricellular proteins on adhesive properties of SMCs in vitro. Although responses varied among replicates, rapamycin treatment did not significantly inhibit the expression of CTGF, thrombospondin-1, or tenascin-C despite marked suppression of S6 activation (Figure 7, A and B). Inhibition of phospho-S6 was also evident when normalized to total S6 levels (Figure 7C). AngII-induced, rapamycin-insensitive CTGF localized to SMCs among other vascular wall cells (Supplemental Figure 5). Since matricellular proteins modulate the adhesion of vascular cells to structural ECM components, which may determine the initiation and propagation of aortic dissections, we investigated this property in vitro. An assay for the adhesion of cultured SMCs to defined ECM proteins was adapted from prior studies of endothelial cells (19). Low-passage SMCs were plated onto plastic precoated with fibronectin, allowed to adhere, washed, and the remaining cells estimated by a colorimetric assay. AngII and rapamycin did not directly affect SMC adhesion to fibronectin (Figure 7D). Treatment of SMCs with recombinant matricellular proteins prior to plating revealed a significant counteradhesive effect of CTGF but not of thrombospondin-1 or tenascin-C at the same doses (Figure 7E). We also measured adhesion of SMCs to plastic coated with matricellular proteins compared with uncoated plastic. In these assays, CTGF but not thrombospondin-1 or tenascin-C directly mediated adhesion of SMCs. Cell binding to CTGF was at least partially blocked by an antibody to integrin α_5_, a known receptor subunit for fibronectin (Figure 7, F and G). Since multiple matricellular proteins are induced by AngII in the vascular wall, we further assessed additive or synergistic interactions in SMC adhesion to fibronectin-coated plates. Neither thrombospondin-1 nor tenascin-C affected the counteradhesive properties of CTGF or modulated each other’s effects (Supplemental Figure 6). Translational relevance of the murine studies was demonstrated by similar CTGF counteradhesive effects on human aortic SMCs from 3 individuals (Figure 7H). These data suggest that the induction of CTGF by AngII is resistant to mTOR inhibition, and CTGF may play a nonredundant role in decreasing SMC adhesion to structural ECM, thus allowing extensive dissection.

## Discussion

Continuous high-rate infusion of AngII for up to 7 days frequently led to aortic tears, adventitial containment of hematomas, with minimal or no dissection, and associated pseudoaneurysm, but seldom led to transmural rupture with associated hemorrhagic mortality and never caused, in 42 infused adult male *Apoe*^–/–^ mice, isolated true dissection of the aorta. These observations merit a revised interpretation of this widely adopted model of aortopathy to define clinical relevancy. AngII-induced aortic disease in *Apoe*^–/–^ mice was first interpreted as abdominal aortic aneurysm based on external expansion of the lesion (9), noting that the term aneurysm derives from the Greek *ανευρυσμα*, meaning “a widening.” This observed aortic pathology was subsequently interpreted as an initial aortic dissection preceding aneurysm formation (10), although without blood within the media. More recently, it has been suggested that this model represents aortic dissection not aneurysm (11), noting that the word dissection comes from the Latin *dissecare*, meaning “to cut apart.” Conversely, pseudoaneurysm formation has been reported (20) and obliquely referred to or conflated with aneurysm and dissection by other investigators (21–23). Herein, we suggest a need for care in the usage of such terms, which are used differently by different communities, but nevertheless should imply consistent meaning. We further suggest that clinical standards be used henceforth since they convey risk, particularly within the purview of clinicians deciding approaches for patient care. Hence, we take aneurysm to denote luminal dilatation of 50% or more and dissection to involve separation of medial layers with intralamellar accumulation of blood. Intimomedial tears are disruptions of the intima that extend into the media for varying depths; full-thickness intimomedial tears sparing the adventitia are considered partial ruptures as often seen in aortic trauma, whereas transmural rupture is a total loss of structural integrity resulting in extravascular hemorrhage. Importantly, pseudoaneurysm refers to a lesion where a large hematoma is contained either by the adventitia after a partial rupture or by the surrounding perivascular tissues adjacent to a transmural rupture. Often in communication with the nonaneurysmal true lumen, contained hematoma causes an overall widening of the blood vessel. Within this lexicon, many have reported histological cross-sectional images of typical lesions similar to Figure 1, C and D, with false lumens, including thrombus between the external elastic laminae and the adventitia (10–15). Prior imaging studies have similarly documented a decrease in true lumen area consistent with Figure 3, A, D, and E (compare with refs. 21, 24), but vascular compartmental areas had not been compared in aortas with and without pseudoaneurysms. The present data emphasize that these hemorrhagic lesions are full-thickness intimomedial tears leading to pseudoaneurysms. Other investigators noted the absence of true dissection within the media in different mouse backgrounds and speculated that the abdominal aorta consists of too few elastic laminae for this complication to occur (25). Yet, the ascending aorta of mice readily dissects in response to AngII as well as in other disease models (18, 26, 27), and our experiments with mTOR inhibition demonstrated that dissection is possible within the suprarenal abdominal aorta. AngII-induced dissection of mouse aortas during rapamycin treatment histologically resembles intramural hemorrhage and not classic dissection, although we have avoided the use of clinical descriptors with particular pathophysiological associations for experimental lesions.

Although definitions of aneurysm and dissection vary in the literature, the concept that “true” aneurysms are produced by dilatation of an artery and “false” aneurysms result from rupture of an artery has been recognized for centuries based on postmortem examinations and for millennia from clinical observations (28). We acknowledge disparities in the definition of rupture and pseudoaneurysm, which may include or exclude the adventitia and subadventitial hematoma, respectively (29). Yet, the potential for the adventitia to contain hematomas and form pseudoaneurysms after traumatic rupture of the aorta is well documented in pathological studies (7, 30, 31). Indeed, damage to the adventitia in penetrating injuries of the aorta may explain persistent bleeding and rare pseudoaneurysm formation compared with blunt injuries of the aorta where preservation of the adventitia is associated with less treacherous bleeding and more stable pseudoaneurysms (31, 32). Even without pseudoaneurysm formation, the adventitia can prevent exsanguination in classic and variant forms of dissection despite marked attenuation sufficient to allow visualization of blood flow and leakage of exudative fluid (2). An alternative interpretation of aortic hematomas after AngII infusion in *Apoe*^–/–^ mice is that it represents a variant form of dissection at the medial-adventitial interface with sparing of the media. Nonetheless, the 280% larger external diameter of the lesion compared with the initial vessel size in our study supports a description as pseudoaneurysm, not variant dissection. Of relevance, clinical dissections within the media acutely increase aorta diameter on the order of 12% (33). Although histology is not used in patients to adjudicate which vascular layer contains extravasated blood, an acute, large, eccentric lesion containing contrast on imaging studies is interpreted as pseudoaneurysm.

The difference between pseudoaneurysm and dissection is not mere semantics. As previously mentioned, a contained rupture of the abdominal aorta is usually a clinical emergency requiring immediate intervention to prevent a high likelihood of death; a dissection of the abdominal aorta, on the other hand, is often observed long-term with medical management of hypertension (8, 34). Although the mechanisms of the two conditions overlap some, namely, an initiating breach of the intima extending into the media, there are important differences. Pseudoaneurysms can enlarge with continued bleeding into the false lumen, increasing the space contained by the remodeling adventitial collagen and perivascular tissues. Reactive adventitial thickening likely explains why free rupture and death seldom occur after 7 days of AngII infusion (16, 22). It is thus likely that mechanisms of pseudoaneurysm enlargement, not those for dissection, lead to the progressive widening of the suprarenal abdominal aorta but not the lumen in this model over 4 weeks (21). This relatively long duration of observation was initially selected to assess effects of AngII on atherosclerosis development, hence the *Apoe*^–/–^ background (9). Many subsequent studies used the same experimental period to investigate mechanisms of abdominal aortic aneurysms and dissections (15). Yet, pseudoaneurysm means “false aneurysm” or in simple language “not an aneurysm.” Therefore, findings in these studies should be reinterpreted as pertaining to pathogenesis of rupture of nonaneurysmal aortas and pseudoaneurysms. Inaccurate description of the lesions as aneurysms, dissections, or dissecting aneurysms (an anachronistic synonym for dissection) is not appropriate to conclude that mechanisms in this model are clinically relevant for aortic aneurysm and dissection, which — except for common intimomedial tears — have very different pathobiology and biomechanics.

We elected to study acute aortic complications induced by AngII within a short period of 7 days when recent mural hematomas remain grossly visible. This strategy ensured targeted examination of aortic segments with hemorrhagic complications, although not necessarily at the site of rupture. Although every hematoma revealed a pseudoaneurysm, concurrent dissection seldom occurred in association with intimomedial tears. Targeted examination of aortas without hematomas was not possible, and limited vascular wall injuries could have been missed by not circumferentially exposing the aorta. After the typical longer duration of AngII infusion for several weeks, smaller hematomas may have resolved, albeit with stigmata of fibrosis and hemosiderin deposition (35). Around half of the animals have rupture or pseudoaneurysm by 7 days, and new lesions uncommonly develop later in the disease course, explaining an absence of fresh hematomas at day 28 of treatment (22). A previous study that closely sectioned the suprarenal abdominal aortas of AngII-treated *Apoe*^–/–^ mice at 28 days regardless of macroscopic appearance found healed full-thickness medial defects (mean of 2.5 tears per aorta) characterized by disrupted elastic lamellae near the origins of major arterial branches (36). These lesions were more numerous but had less collagen in aortas with pseudoaneurysms than without. A few such injuries of lesser extent, however, were also found in saline-treated *Apoe*^–/–^ mice (mean of 0.7 tears per aorta), supporting a hypothesis that intimomedial tears can heal without significant sequelae. Imaging-guided analyses confirmed limited intimomedial tears infiltrated with contrast agent, termed “micro-ruptures,” at arterial orifices as early as 3 days after initiation of AngII infusion (11). By 28 days, all aortas displayed intimomedial tears, the majority associated with subadventitial hematomas, termed “adventitial dissections,” and less commonly the full-thickness tears of the media were simply covered by the adventitia without bleeding but resulted in focal dilatations of the vascular wall. Although the findings were interpreted as causative of aortic dissection, medial hematomas were not evident by histology. In a follow-up study, interlamellar bleeding was found in artery branches and minimally within the outermost medial lamella extending into the adventitia, termed “intramural hematoma” (37), but not overt dissection as seen in the ascending aorta of wild-type mice after AngII infusion (26, 27) or in the suprarenal abdominal aorta of *Apoe*^–/–^ mice with concomitant rapamycin treatment. We did not find evidence for medial degeneration after 1–7 days of AngII infusion, and there were few elastin breaks far from the sites of intimomedial tears. In a previous longer-term study, we found no loss of elastin or accumulation of glycosaminoglycans in the suprarenal aorta of *Apoe*^–/–^ mice after 4–28 days of AngII infusion (16). Thus, intimomedial tears can be triggered by increased wall stress without the preexistent medial degeneration characteristic of aging and chronic aortic diseases. A corollary is that AngII infusion in *Apoe*^–/–^ mice is not a suitable model for medial degeneration.

The predisposition for aortic rupture or dissection in AngII-treated murine aortas without and with mTOR inhibition, respectively, implies differential vulnerabilities of the vascular wall. As discussed above, radial tears of the aorta may extend through the full thickness of the media. The thin adventitia consisting of tightly woven collagen is nevertheless sufficient to prevent free rupture and exsanguination in most cases. Adhesion of the adventitia to the external elastic lamina is usually insufficient, however, to prevent mural accumulation of blood and pseudoaneurysm formation. Notably, genetic absence of thrombospondin-2 predisposes to delamination at this same interface (38), suggesting the importance of matricellular proteins at lamellar interfaces. Toward this end, conversion of aortic rupture to dissection by rapamycin suggests that adhesion of SMCs to the ECM may play a key role in the structural integrity of the media. Contractile forces exerted by SMC on structural ECM components of the media prevent aortic dissection in another model of thoracic aortopathy and are modulated by rapamycin treatment (39). Of relevance, we found that mTOR inhibition did not diminish the rapid induction of matricellular proteins, nonstructural ECM components that also modulate the adhesion of endothelial cells to other ECM molecules such as fibronectin in vitro (19, 40). The dynamic regulation of this class of ECM molecules correlates with the earliest lesion occurrence at 1 day. Hence, pathological roles for these matricellular proteins cannot be excluded in diminishing endothelial cell adhesion and initiating intimal tears. We focused, however, on the media, and our in vitro experiments suggest that CTGF has a dominant counteradhesive effect on SMC interactions with ECM, including human cells. One possible mechanism is via competitive binding to integrin α_5_, a receptor subunit for fibronectin; CTGF binds to this integrin in other cell types (41). It appears, therefore, that matricellular-mediated effects on SMC adhesion to the ECM can compromise intralamellar integrity. Conversely, CTGF has profibrotic effects, which may strengthen the media and adventitia depending on timing and context. Previous experiments demonstrated that genetic absence of thrombospondin-1 or tenascin-C worsened AngII-mediated aortopathy, but these matricellular proteins may not be relevant for SMCs and medial defects, and these studies used germline deficiency models in which altered vascular wall development and effects on nonvascular cell types cannot be distinguished from acute effects on vascular cell adhesion; moreover, effects on deposition and degradation of structural ECM, not cellular adhesion, were investigated, and chronic readouts of lesion diameter were assessed rather than aortic tears (25, 42). Although we did not investigate the effects of rapamycin with saline infusion, other investigators have not reported aortic dissections in *Apoe*^–/–^ mice treated with rapamycin alone (43, 44), and it is relevant that the expression of matricellular proteins within the mature aorta is not increased in the absence of AngII infusion.

Finally, we found an association of inflammation and production of MMPs with aortic rupture, consistent with previous causal roles for these biological processes in AngII-mediated aortopathy (45, 46). Others have documented that rapamycin decreases the incidence of AngII-mediated aortopathy by preventing macrophage recruitment and activation and thus reducing MMP production (47, 48). Yet, the early occurrence of some lesions within 1 day of AngII infusion suggests that not all aortic rupture is mediated by monocyte recruitment, although a role for sparse tissue-resident macrophages cannot be excluded. The *Apoe*^–/–^ mutation may predispose to aortic rupture from underlying vascular inflammation and compromised adventitial integrity. Since adventitial collagen buttresses the external elastic lamina, the strength of the outer media and the occurrence of full-thickness intimomedial tears may also depend on perivascular macrophage activity. By suppressing macrophage responses, rapamycin prevents aortic rupture and pseudoaneurysm but not dissection resulting from direct effects of AngII on SMC. Rapamycin may also modulate aortopathy via SMC effects, and we have previously shown that SMC contraction prevents medial delamination of vulnerable aortas ex vivo (39). Although mTOR inhibition increases contractile molecule expression in cultured (dedifferentiated) SMCs and in elastin-deficient thoracic aortas in vivo (49, 50) and improves contractility of aortas with disrupted TGF‑β signaling over 4 weeks (39), we did not find that short-term treatment with rapamycin for 7 days improved the reduced contractility of suprarenal abdominal aortas exposed to AngII infusion (compare with Figure 6B). Delineation of macrophage- versus SMC-dependent mechanisms in AngII-induced aortic rupture and dissection requires further genetic models for cell-conditional disruption of mTOR signaling.

In conclusion, dissection and rupture represent a continuum of aortic disease with common precursor lesions of intimomedial tears and varying manifestations dependent on mTOR activity of vascular wall cells. Subtleties of aortic pathology should not be oversimplified given that convenient readouts and precise identification of histopathological findings in experimental models are necessary for appropriate translation to clinical disease. Although mTOR inhibition prevents aortic rupture and pseudoaneurysm, the emergence of dissection necessitates caution regarding possible clinical use, with a pressing need to better understand the roles of matricellular, not only structural, proteins in aortic health and disease.

## Methods

### Mice.

Male *Apoe*^–/–^ mice on a C57BL/6J background (002052, Jackson Laboratory), fed a regular laboratory diet, were studied at 12 weeks of age (*n* = 145) to maintain uniform aorta maturation and size. These mice were treated with saline, AngII (Sigma-Aldrich) at 1000 ng/kg/min s.c. as a constant infusion, and/or rapamycin (Calbiochem) at 2 mg/kg/d i.p. every day for varying durations, and the suprarenal abdominal aortas were characterized using multiple approaches (Supplemental Table 3). In most experiments, implantation of s.c. pumps and initiation of AngII infusions was at 11 weeks of age and the animals were euthanized at 12 weeks of age (termed 7-day infusion). In certain experiments, s.c. pump implantation and AngII infusion initiation were 1 or 3 days prior to euthanasia at 12 weeks of age (termed 1- and 3-day infusions, respectively). Rapamycin treatment generally started at the time of AngII infusion initiation and continued until euthanasia (termed day 0 to 1–7 treatment), except in 1 subgroup in which rapamycin treatment started 2 weeks prior to AngII infusion initiation (termed day –14 to 7 treatment). Key findings of AngII-induced aortic lesions and mTOR signaling in SMCs were confirmed in additional mice: 12-week-old female mice (*n* = 6) and 3-week-old male mice (*n* = 6).

### Pump implantation.

Osmotic pumps (1003D and 1007D, Alzet) were primed with saline or AngII overnight prior to implantation. Using a sterile technique and under inhaled isoflurane anesthesia (2% at 1 L/min), an incision was made on the dorsum, the subcutaneous tissue was bluntly dissected, the pump implanted, and the incision closed with 6/0 nonabsorbable suture. Buprenorphine (0.1 mg/kg i.p. or s.c.) was administered preoperatively and every 12 hours for 48 hours postoperatively.

### In situ examination.

After ketamine (100 mg/kg i.p.) and xylazine (10 mg/kg i.p.), a midline incision was performed, the abdomen and chest were opened widely, the circulation was flushed with saline, the viscera were excised, and the retroperitoneum entered. The suprarenal abdominal aorta was inspected for hematomas using a SZX16 dissecting microscope with a camera attachment (Olympus).

### Blood pressure and ultrasound.

Blood pressure was measured noninvasively in conscious animals using a CODA volume-pressure recording sensor and occlusion tail-cuff (Kent Scientific Corporation). Mice were placed in warmed restraining chambers and pressures were recorded for 10 cycles after discarding the first 5 data points. Transabdominal B-mode images of the suprarenal abdominal aorta were obtained in lightly isoflurane anesthetized animals using a Vevo 770 high-frequency ultrasound (VisualSonics). Inner-to-inner true lumen and inner true lumen to outer false lumen diameters were measured.

### Histology.

The aorta was excised from diaphragm to renal arteries, postfixed in 4% paraformaldehyde at 4°C overnight, transferred to 70% ethanol at 4°C for 24–72 hours, and then embedded in paraffin with the blocks oriented transversely for most specimens and longitudinally for 3 specimens with hematomas. Next, 5 μm thick sections were stained with H&E or Verhoeff-Van Gieson by Yale’s Research Histology Laboratory using standard techniques and an automated system.

### Histomorphometry and cell counts.

Morphometry was performed using ImageJ (NIH) after outlining the internal and external elastic laminae and the outer adventitial perimeter. Lumen area (including the negligible intimal area) was calculated within the internal elastic lamina. Media area was calculated between the internal and external elastic laminae. Adventitia area was calculated between the external elastic laminae and the outer adventitial perimeter; this area included extravasated blood in pseudoaneurysms. The adventitia was defined as the dense fibrous tissue adjacent to the external elastic lamina, whereas perivascular tissue was considered as the loose connective tissue external to the adventitia. The number of medial cells was calculated by counting hematoxylin-stained nuclei between the internal and external elastic laminae. The number of F4/80^+^ macrophages was counted separately in the vascular wall compartments, including the perivascular tissue.

### IHC, fluorescence microscopy, biomechanical assessment, flow cytometry, Western blot, quantitative RT-PCR, and cell culture.

Descriptions are provided in the Expanded Methods of the Supplemental Material.

### Adhesion assays.

First, 96-well flat-bottom plates (351172, Falcon) were coated with purified bovine fibronectin (150025, MP Biomedicals) at 6 μg/mL in 50 μL PBS at 4°C overnight. The plates were washed once with PBS, blocked with freshly prepared, heat-denatured BSA (A9418, Sigma-Aldrich) at 10 mg/mL dissolved in PBS for 1 hour, and washed twice. Early-passage SMCs were trypsinized, the enzymatic activity stopped with soybean trypsin inhibitor, and cells were resuspended in serum-free DMEM containing 1 mg/mL BSA. In certain experiments, the cells were pretreated with vehicle, AngII (A9525, Sigma-Aldrich) at 100 nM, or rapamycin (553210, Sigma-Aldrich) at 100 ng/mL; in other experiments, the cells were pretreated with CTGF, thrombospondin-1, or tenascin-C at 12.5 or 25 μg/mL, alone or in various combinations, for 45 minutes at 37°C. The cells were seeded at 3 × 10^4^ cells in 100 μL solution per fibronectin-coated well, incubated for 1 hour at 37°C, and washed twice with PBS containing 1 mM Ca^2+^ and 2 mM Mg^2+^. Alternatively, for fibronectin-independent adhesion assays, the plates were coated with recombinant human CTGF (9190-CC-050, R&D Systems), mouse thrombospondin-1 (17859-TH-050, R&D Systems), or human tenascin-C (3358-TC-050, R&D Systems) at 0.5–50 μg/mL; adhesive properties of human matricellular proteins were validated as cross-reactive on murine cells by the supplier. In certain experiments with CTGF-coated plates, the cells were pretreated with blocking antibody to integrin α_5_ (103910, BioLegend) or isotype-matched control antibody (400940, BioLegend) at 10 μg/mL. The number of adherent cells was determined by a colorimetric assay in which 100 μL of nitrophenyl phosphate (P4744, Sigma-Aldrich) at 3 mg/mL in 50 mM sodium acetate, pH 5.0, plus 0.4% Triton X-100 was added to each well and incubated at room temperature for 1 hour; then, 50 μL NaOH at 1M was added to each well and the OD was determined spectrophotometrically at 405 nm. OD values were corrected for empty well readings and when pooled from several experiments, the results were normalized to untreated (fibronectin alone) controls because of batch variability.

### Statistics.

Quantitative data are presented as dot plots with bars representing the mean **±** SEM. Repeated observations are presented as means with connecting lines and shaded error bars representing SEM. Single numerical values are represented by columns. Survival curves show the percentage of surviving animals over time based on daily observations. Comparison of continuous variables between 2 groups was by 2-tailed Student’s *t* test, between more than 2 groups with 1 independent variable by 1-way ANOVA followed by Tukey’s multiple-comparison test, or with 2 independent variables by 2-way ANOVA followed by Sidak’s multiple-comparison test. Comparison of categorical variables was by Fisher’s exact test. Probability values were 2‑tailed and *P* less than 0.05 was considered to indicate statistical significance. Graph construction and statistical analyses were performed with GraphPad Prism 8.2.0.

### Study approval.

Animal research protocols were approved by the IACUC of Yale University. Research protocols for human subjects were approved by the IRBs of Yale University and the New England Organ Bank, with a waiver for patient consent.

## Author contributions

CH, BJ, and GT designed the study. CH, BJ, SIM, MW, PR, and AWC conducted experiments and acquired data. CH, BJ, SIM, JDH, and GT analyzed and interpreted data. GL, LQ, JDH, and GT supervised the work. CH, BJ, RA, CJL, MAS, JDH, and GT wrote and edited the manuscript. The order of the co–first authors was determined by the chronology of the effort: CH initiated the work and BJ completed the work, with both collectively performing the bulk of the work and making critical contributions.

## Supplementary Material

Supplemental data

## Version 1 02/08/2022

Electronic publication
