# Supplementary material for: mTOR inhibition prevents angiotensin II–induced aortic rupture and pseudoaneurysm but promotes dissection in Apoe-deficient mice
Source: JCI Insight. 2022 Feb 8;7(3):e155815. doi: 10.1172/jci.insight.155815 (PMC8855820; doi:10.1172/jci.insight.155815)
Supplement: Supplemental data [file jciinsight-7-155815-s166.pdf]

SUPPLEMENTAL MATERIAL

mTOR Inhibition Prevents Angiotensin II-Induced Aortic Rupture and Pseudoaneurysm but Promotes Dissection in *Apoe*-Deficient Mice

Changshun He[1,2,*], Bo Jiang[1,3,*], Mo Wang[1], Pengwei Ren[1], Sae-Il Murtada[4], Alexander W. Caulk[4], Guangxin Li[1,5], Lingfeng Qin[1], Roland Assi[1,6,7], Constantinos J. Lovoulos[7,8], Martin A. Schwartz[3,9,10,11], Jay D. Humphrey[4,6], George Tellides[1,6,7]

[1]Department of Surgery (Cardiac), Yale School of Medicine, New Haven, CT, USA.

[2]Current affiliation: Department of Vascular Surgery, Peking University People's Hospital, Beijing, China.

[3]Current affiliation: Department of Vascular Surgery, The First Hospital of China Medical University, Shenyang, Liaoning, China.

[4]Department of Biomedical Engineering, Yale School of Engineering and Applied Science, New Haven, CT, USA.

[5]Current affiliation: Department of Breast and Thyroid Surgery, Peking University Shenzhen Hospital, Shenzhen, Guangdong Province, China.

[6]Program in Vascular Biology and Therapeutics, Yale School of Medicine, New Haven, CT, USA.

[7]Veterans Affairs Connecticut Healthcare System, West Haven, CT, USA.

[8]Department of Surgery, Frank H. Netter MD School of Medicine, Quinnipiac University, North Haven, CT, USA.

[9]Department of Medicine (Cardiology), Yale School of Medicine, New Haven, CT, USA.

[10]Department of Cell Biology, Yale School of Medicine, New Haven, CT, USA.

[11]Yale Cardiovascular Research Center, Yale School of Medicine, New Haven, CT, USA.

*Denotes co-first authorship.

Expanded Methods

Immunohistochemistry

Five µm-thick sections were deparaffinized, rehydrated with distilled water, placed in Tris-buffered saline with 0.1% Tween 20, and labeled with an antibody to F4/80 (14-4801-81, Thermo Fisher), TER-119 (116202, Biolegend), or isotype-matched, nonbinding immunoglobulin. Binding of secondary antibodies was detected with the HRP-based EnVision+ System and Liquid DAB+ Substrate-Chromogen kit (DAKO), counter-stained with hematoxylin (Sigma-Aldrich), and imaged using an Axioskop2 microscope (Carl Zeiss MicroImaging).

Immunofluorescence Analysis

Five µm-thick sections were incubated with FITC-conjugated antibody to SMA (F3777, Sigma-Aldrich) or antibodies to thrombospondin-1 (MA5-13395, Invitrogen), tenascin-C (MA1-26778, Invitrogen), CTGF (PA1-22376, Invitrogen), and isotype-matched, irrelevant IgG overnight at 4 °C followed by Alexa Fluor 488- or 647-conjugated secondary antibody (Invitrogen) for 1 hour at room temperature. The slides were incubated with Pro-Long Gold Mounting Reagent with DAPI (Life Technologies). Immunofluorescence images were acquired using an Axiovert 200M microscopy system (Carl Zeiss MicroImaging).

Confocal Microscopy

Five µm-thick sections were incubated with FITC-conjugated mouse monoclonal antibody to SMA (F3777, Sigma-Aldrich), or unconjugated antibodies of goat anti-mouse CD31 (AF3628, R&D), rat anti-mouse TER-119 (116202, Biolegend), rabbit anti-mouse CD45 (70257S, Cell Signaling), rabbit anti-phospho-S6-S235/236 (4858, Cell Signaling), and rabbit anti-CTGF (PA1-22376, Invitrogen) overnight at 4 °C followed by Alexa Fluor 488- or 568-conjugated IgG (Invitrogen). The sections were covered with Alexa Fluor 633 hydrazide dye specific for elastin

(A30634, Thermo Fisher) for 10 min at room temperature and then mounted with ProLong Gold Antifade reagent with DAPI (Life Technologies). Images were acquired with a SP8 confocal microscope (Leica).

Biomechanical Assessment

Tissue Preparation: Biaxial mechanical tests and data analysis were performed on excised segments of the suprarenal abdominal aorta from *Apoe*$^{-/-}$ mice at 12 weeks of age treated for 7 days with saline, AngII, or AngII and rapamycin. The aortic segments were gently cleaned of excess perivascular tissue and branches were ligated with 9-0 nylon suture. The excised vessels were cannulated on custom-drawn glass pipets, secured with sutures at each end, and mounted on a biaxial testing device, submerged in Krebs-Ringer's solution at 37 °C and oxygenated with 95% O_2 / 5% CO_2.

Active Mechanical Contraction Testing: The viability of the active tone in the aorta was assessed through two initial contractions to 100 mM KCl at two different combinations of pressures and vessel lengths followed by washouts with normal Krebs-Ringer solution. The vessels were then set to 90 mmHg and in vivo value of axial stretch and contracted with 100 mM KCl for 15 minutes followed by 10 minutes of relaxation by washout. This was repeated for 1 μM phenylephrine and, before washing out, 10 μM acetylcholine was added to assess endothelial function.

Passive Mechanical Biaxial Testing: After completing the active testing, the testing chamber was drained and refilled with Hanks' buffered salt solution and maintained at room temperature to minimize smooth muscle contractility. Vessels were mechanically preconditioned, by cyclic pressurization between 10 to 140 mmHg at the estimated in vivo value of axial stretch, to minimize viscoelastic contributions to the mechanical behavior. The aortic segments were then subjected to a series of seven biaxial protocols consisting of cyclic pressurization from 10 to 140 mmHg

while the vessel was held fixed at three different axial stretches (95, 100, and 105% of the in vivo value), and cyclic axial stretching at four fixed pressures (10, 60, 100, and 140 mmHg).

Data Analysis of Active and Passive Mechanical Properties: The contractile properties were assessed by calculating changes in inner radius and mean circumferential stress between relaxed and contracted states. The passive pressure-diameter and axial force-length data were fit with a validated four-fiber family constitutive model via a nonlinear regression of a data set from all seven testing protocols. Specifically, we used a Holzapfel-type nonlinear stored energy function *W*,

$$W\left(\mathbf{C},\mathbf{M}^{i}\right)=\frac{c}{2}\left(I_{\mathbf{C}}-3\right)+\sum_{i=1}^{4}\frac{c_{1}^{i}}{4c_{2}^{i}}\left\{\exp\left[c_{2}^{i}\left(IV_{\mathbf{C}}^{i}-1\right)^{2}\right]-1\right\}\ , \qquad (xx)$$

where c, c_1^i, and c_2^i ($i=1{,}2{,}3{,}4$ denote the four predominant fiber family directions) are material parameters, with c and c_1^i having units of stress (kPa) and c_2^i dimensionless. $I_{\mathbf{C}}=tr(\mathbf{C})$ and $IV_{\mathbf{C}}^i=\mathbf{M}^i\cdot\mathbf{C}\mathbf{M}^i$ are coordinate invariant measures of the deformation, computed in terms of the right Cauchy-Green tensor $\mathbf{C}=\mathbf{F}^{\mathrm{T}}\mathbf{F}$ where the deformation gradient tensor $\mathbf{F}=\mathrm{diag}[\lambda_r,\lambda_\theta,\lambda_z]$, with $det\mathbf{F}=1$ because of assumed incompressibility. The direction of the ith family of fibers is identified by the vector $\mathbf{M}^i=\left[0,\ \sin\alpha_0^i,\ \cos\alpha_0^i\right]$, with the model parameter α_0^i denoting a fiber angle relative to the axial direction in the traction-free reference configuration. Values of biaxial stress and material stiffness were computed from appropriate differentiation of the stored energy function.

Flow Cytometry

Aortic tissue was minced and incubated in 0.5 ml DMEM with 10% FBS, 1.5 mg/ml collagenase A, and 0.5 mg/ml elastase for 60 min at 37°C while the tissue fragments were gently triturated using a pipette every 15 min. The digested aorta solution was passed through a 40 µm filter and incubated with cell-impermeant viability dye (65-0865-14, Thermo Fisher) for 20 min,

fixed with Fixation Buffer (eBioscience) for 15 min, washed, resuspended in Permeabilization Buffer (eBioscience), and blocked with unconjugated Fc antibodies (Biolegend) for 20 min, all at 4°C. The cells were stained with fluorescent-labeled antibodies to SMA (F3777, Sigma-Aldridge), CD11b (562605, BD Biosciences), CD31 (102515, BioLegend), or isotype-matched, irrelevant IgG for 1 h on ice in the dark. The cells were pelleted and resuspended in 0.4% BSA/PBS for analysis using a LSR II (BD Biosciences). Sequential gating was used to exclude: i) small debris and damaged cells by forward versus side scatter area, ii) doublets by side scatter followed by forward scatter width versus height, and iii) non-viable cells by impermeant dye uptake. Forward scatter area was used as an indicator of cell size and side scatter area was used as an indicator of cell granularity. The data were analyzed with FlowJo software.

Western Blot

Protein was extracted from homogenized frozen aortas without hematomas using RIPA lysis buffer containing protease and phosphatase inhibitor cocktail tablets (Roche) and boiled in SDS sample buffer for 5 min. Equal amounts of protein per sample were separated by SDS-PAGE, transferred electrophoretically to a PVDF membrane (Bio-Rad Laboratories), and blotted with antibodies to phospho-S6-S235/236 (4858, Cell Signaling), S6 (2217, Cell Signaling), CTGF (PA1-22376, ThermoFisher), thrombospondin-1 (37879, Cell Signaling), tenascin-C (12221S, Cell Signaling), or HSP90 (SAB4300541, Sigma-Aldrich), followed by horseradish peroxidase-conjugated secondary antibodies. The blotting membrane was cut to allow detection of proteins of different molecular weights. Bound antibody was detected with Western Lightning Plus-ECL (Perkin Elmer) using autoradiography film for shorter exposures or an imaging system (ChemiDoc MP Imaging System, Bio-Rad) for longer exposures.

Quantitative RT-PCR

Frozen aortas without hematomas were crushed and the tissue fragments were immersed in RLT lysis buffer (Qiagen) and vigorously vortexed. Total RNA was isolated using a RNeasy Mini Kit and DNase Digestion Set (Qiagen) according to the manufacturer's protocol. Reverse transcriptions were performed using an iScript cDNA Synthesis Kit (Bio-Rad). Quantitative RT-PCR was performed using a real-time PCR detection system (CFX96, Bio-Rad) by mixing equal amount of cDNAs, Taqman gene master mix, and primers from Applied Biosystems for *Col1a1* (Mm00801666_g1), *Col3a1* (Mm00802300_m1), *Spp1* (Mm00436767_m1), *Mmp2* (Mm004394-98_m1), *Mmp3* (Mm00440295_m1), *Mmp14* (Mm00485054_m1), *Thbs1* (Mm00449032_m1), *Tnc* (Mm00495662_m1), *Ccn2* (Mm01192933_g1), and *Gapdh* (Mm99999915_g1). RNA-free ddH2O was used as negative controls for qPCR instead of cDNA samples. All reactions were in a 12.5 µl volume, in duplicate. PCR amplification consisted of 10 min of an initial denaturation step at 95 °C followed by 40 cycles of PCR at 95 °C for 15 s, and 60 °C for 1 min. We confirmed stable expression of a second housekeeping gene, *Hprt1* (Hs02800695_m1).

Cell Culture

Murine SMCs were derived by explant outgrowth from minced fragments of thoracic aortas from C57BL/6J mice. Human SMCs were derived by explant outgrowth from medial fragments of non-diseased ascending aortas of 3 organ donors whose hearts were not used for clinical transplantation. The cells were cultured in DMEM with 10% FBS and used between passage 1-3 for mouse SMC and passage 4-5 for human SMC.

Supplemental Table 1: Summary of passive biomechanical data in numerical form*

	**Saline** *n* = 5	**AngII** *n* = 4	**AngII+Rapa** *n* = 5
**Unloaded dimensions**			
Outer diameter (μm)	823 ± 16	890 ± 39	875 ± 27
Wall thickness (μm)	104 ± 4	126 ± 3	131 ± 6
Axial length (mm)	5.70 ± 0.30	6.15 ± 0.52	5.70 ± 0.21
**Loaded dimensions at systole**	**P = 108**	**P = 149**	**P = 146**
Outer diameter (μm)	1311 ± 32	1551 ± 34	1427 ± 62
Wall thickness (μm)	35.6 ± 1.6	41.0 ± 2.8	52.6 ± 3.2
Inner radius (μm)	620 ± 15	735 ± 15	661 ± 31
Axial stretch (λ_z^{iv})	1.64 ± 0.02	1.55 ± 0.05	1.35 ± 0.03
Circumferential stretch (λ_ϑ)	1.77 ± 0.02	1.99 ± 0.06	1.85 ± 0.06
**Cauchy stresses (kPa)**			
Circumferential, σ_ϑ	252 ± 10	360 ± 21	247 ± 18
Axial, σ_z	241 ± 18	272 ± 21	174 ± 17
**Linearized stiffness (MPa)**			
Circumferential,C_{qqqq}	1.53 ± 0.08	2.37 ± 0.21	1.80 ± 0.12
Axial, C_{zzzz}	2.07 ± 0.13	2.59 ± 0.09	2.04 ± 0.20
**Stored energy (kPa)**	72.7 ± 4.9	83.1 ± 7.2	49.1 ± 5.3
**Loaded dimensions at 100 mmHg**	**P = 100**	**P = 100**	**P = 100**
Outer diameter (μm)	1286 ± 31	1399 ± 41	1316 ± 54
Wall thickness (μm)	36.4 ± 1.6	45.8 ± 3.1	57.4 ± 3.3
Inner radius (μm)	607 ± 15	654 ± 19	600 ± 26
Axial stretch (λ_z^{iv})	1.64 ± 0.02	1.55 ± 0.05	1.35 ± 0.03
Circumferential stretch (λ_ϑ)	1.74 ± 0.02	1.78 ± 0.04	1.69 ± 0.05
**Cauchy stresses (kPa)**			
Circumferential, σ_ϑ	224 ± 9	192 ± 11	141 ± 9
Axial, σ_z	225 ± 17	177 ± 17	114 ± 11
**Linearized stiffness (MPa)**			
Circumferential,C_{qqqq}	1.28 ± 0.07	1.00 ± 0.10	0.81 ± 0.05
Axial, C_{zzzz}	1.84 ± 0.12	1.43 ± 0.09	1.17 ± 0.09
**Stored energy (kPa)**	67.9 ± 4.7	54.0 ± 4.6	32.4 ± 3.2

***Mean ± SEM values of all geometric and mechanical metrics calculated from the ex vivo passive biomechanical testing, including unloaded dimensions, values representing in vivo conditions at group specific systolic pressures (P), or values at equal pressure, of the suprarenal abdominal aorta of 12-week-old *Apoe*$^{-/-}$ mice treated with saline, AngII (1,000 ng/kg/min s.c.), or AngII and rapamycin (Rapa, 2 mg/kg/d i.p.) for 7 days. See Figures 2 and 5 for graphical representation of individual data for selected parameters.

Supplemental Table 2: Summary of active biomechanical data in numerical form*

	Saline<br>*n* = 5	AngII<br>*n* = 4	AngII+Rapa<br>*n* = 5
**100 mM KCl**			
**Loaded configuration**			
Relaxed inner radius (μm)	569 ± 21	584 ± 27	470 ± 19
Contracted inner radius (μm)	528 ± 21	561 ± 25	455 ± 17
Change in inner radius (%)	-7.3 ± 0.4	-4.0 ± 0.9	-3.1 ± 1.2
**Stress calculations**			
Relaxed circumferential stress (kPa)	159 ± 6	129 ± 6	77 ± 3
Contracted circumferential stress (kPa)	137 ± 5	120 ± 10	73 ± 4
Change in circumferential stress (%)	-13.5 ± 0.7	-7.5 ± 1.7	-5.9 ± 2.0
**1 μM PE**			
**Loaded configuration**			
Relaxed inner radius (μm)	582 ± 22	586 ± 28	468 ± 20
Contracted inner radius (μm)	502 ± 14	544 ± 29	426 ± 16
Change in inner radius (%)	-13.6 ± 1.7	-7.1 ± 1.9	-8.7 ± 3.2
**Stress calculations**			
Relaxed circumferential stress (kPa)	166 ± 6	130 ± 11	76 ± 1
Contracted circumferential stress (kPa)	125 ± 2	113 ± 11	65 ± 4
Change in circumferential stress (%)	-24.4 ± 2.9	-13.1 ± 3.5	-15.1 ± 5.3
**Metric normalized to KCl response**			
Change in circumferential stress (%)	190 ± 21	175 ± 39	280 ± 69
**10 μM ACh + 1 μM PE**			
**Loaded configuration**			
Dilated inner radius (μm)	562 ± 21	581 ± 32	462 ± 20
Change in inner radius (%)	11.9 ± 3.1	6.8 ± 2.3	8.5 ± 3.0
**Stress calculations**			
Dilated circumferential stress (kPa)	155 ± 7	128 ± 11	75 ± 5
Change in circumferential stress (%)	24.4 ± 6.7	13.5 ± 4.7	16.6 ± 5.9
**Metric normalized to PE response**			
Relaxation capability (%)	69.4 ± 12.3	91.1 ± 16.7	103.4 ± 64.9

***Mean ± SEM values of all geometric and mechanical metrics calculated from the ex vivo active biomechanical testing, including loaded dimensions and values in response to KCl (100 mM), phenylephrine (PE, 1 μM), or acetylcholine (ACh, 10 μM) plus phenylephrine, of the suprarenal abdominal aorta of 12-week-old *Apoe*$^{-/-}$ mice treated with saline, AngII (1,000 ng/kg/min s.c.), or AngII and rapamycin (Rapa, 2 mg/kg/d i.p.) for 7 days. See Figure 5 for graphical representation of individual data for selected parameters.

Supplemental Table 3: Number of experimental animals per treatment group*

	Saline<br>*n* = 33	AngII<br>*n* = 59	AngII+Rapa<br>*n* = 53
Microscopy	8	31	28
Biomechanics	5	4	5
Quantitative RT-PCR	4	8	8
Western blot	8	8	4
Flow cytometry	8	8	8

*Suprarenal abdominal aortas of 12-week-old, male *Apoe*$^{-/-}$ mice (*n* = 145) were comprehensively characterized after various pharmacological interventions of saline, AngII, or AngII plus rapamycin (Rapa). The aortic segments were of limited size and each specimen was analyzed using a single technique. Microscopy included histology, histomorphometry, immunohistochemistry, immuno-fluorescence, and confocal procedures. Aortas with hemorrhagic lesions were analyzed by microscopy but excluded from other assays to avoid the confounding influence of extravasated blood. Additional cohorts of 12-week-old, male mice (*n* = 6) used to derive SMC cultures and 12-week-old, female mice (*n* = 6) and 3 week-old, male mice (*n* = 6) used for preliminary signaling studies are not included.

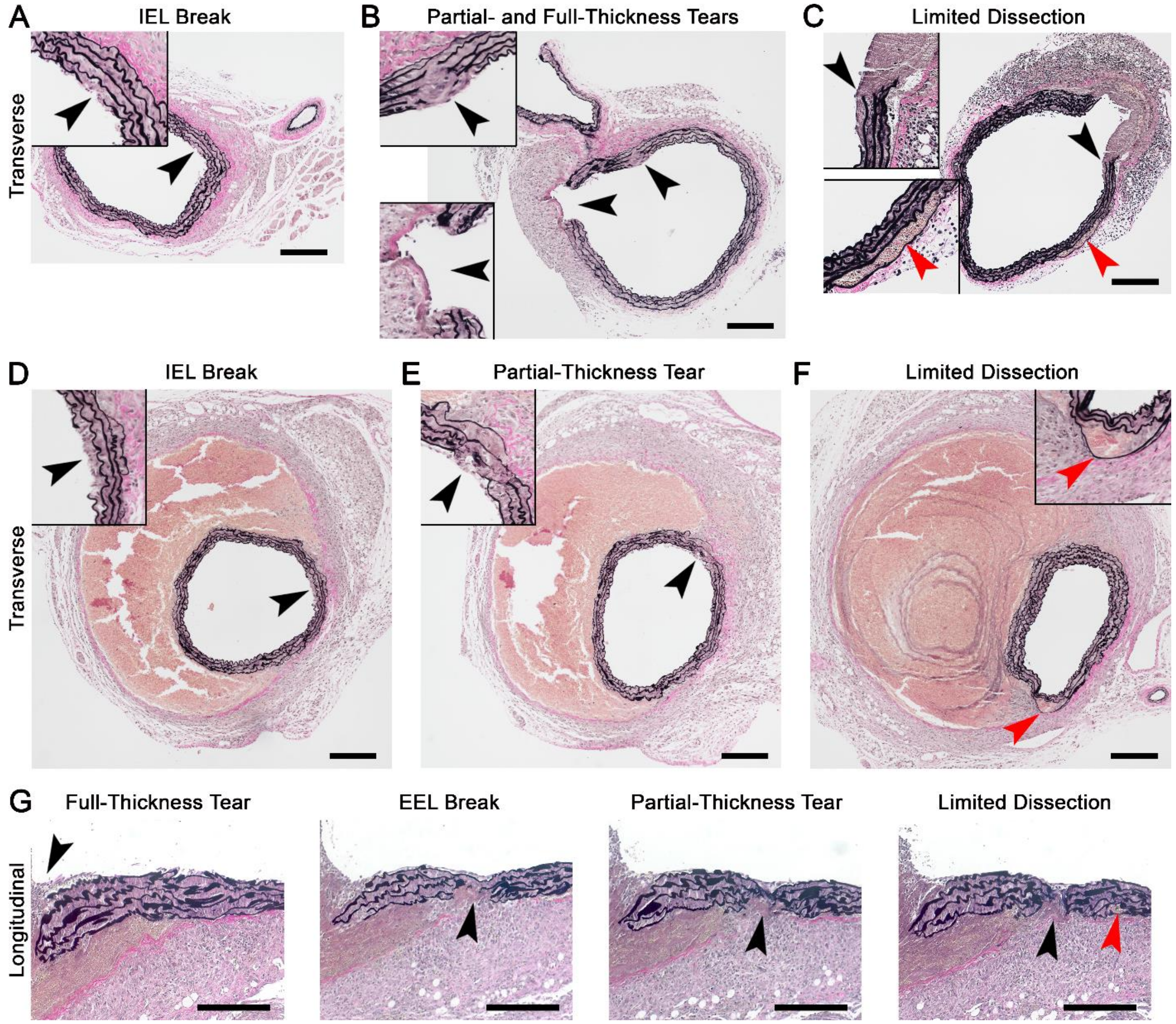

Supplemental Figure 1: AngII-induced intimomedial tears result in a spectrum of aortic complications. Adult male *Apoe*$^{-/-}$ mice were infused with AngII for 7 days and the suprarenal abdominal aortas were fixed in the unloaded state and analyzed by Verhoeff-Van Gieson stain in serial sections to avoid misidentification of artifacts. Transverse sections of aortas without visible hematomas showing (**A**) internal elastic lamina break (black arrowhead), (**B**) partial- and full-thickness intimomedial tears (black arrowheads) without subadventitial hemorrhage, and (**C**) full-thickness intimomedial tear (black arrowhead) with contained rupture and limited medial dissection (red arrowhead). Transverse sections of aortas with gross hematomas showing (**D**) internal elastic lamina break (black arrowhead), (**E**) partial-thickness intimomedial tear (black arrowhead), and (**F**) limited medial dissection (red arrowhead). (**G**) Longitudinal section of aorta with gross hematoma (low magnification view in Figure 2D) showing full-thickness tear with contained rupture (black arrowhead, left panel) and progression of a second intimomedial tear (black arrowheads, right 3 panels) from outer laminae to internal elastic lamina with signs of tissue repair and an associated limited medial dissection (red arrowhead). Composite photomicrographs (panels A-F) with insets showing intimomedial tears at higher magnification, scale bars = 200 μm.

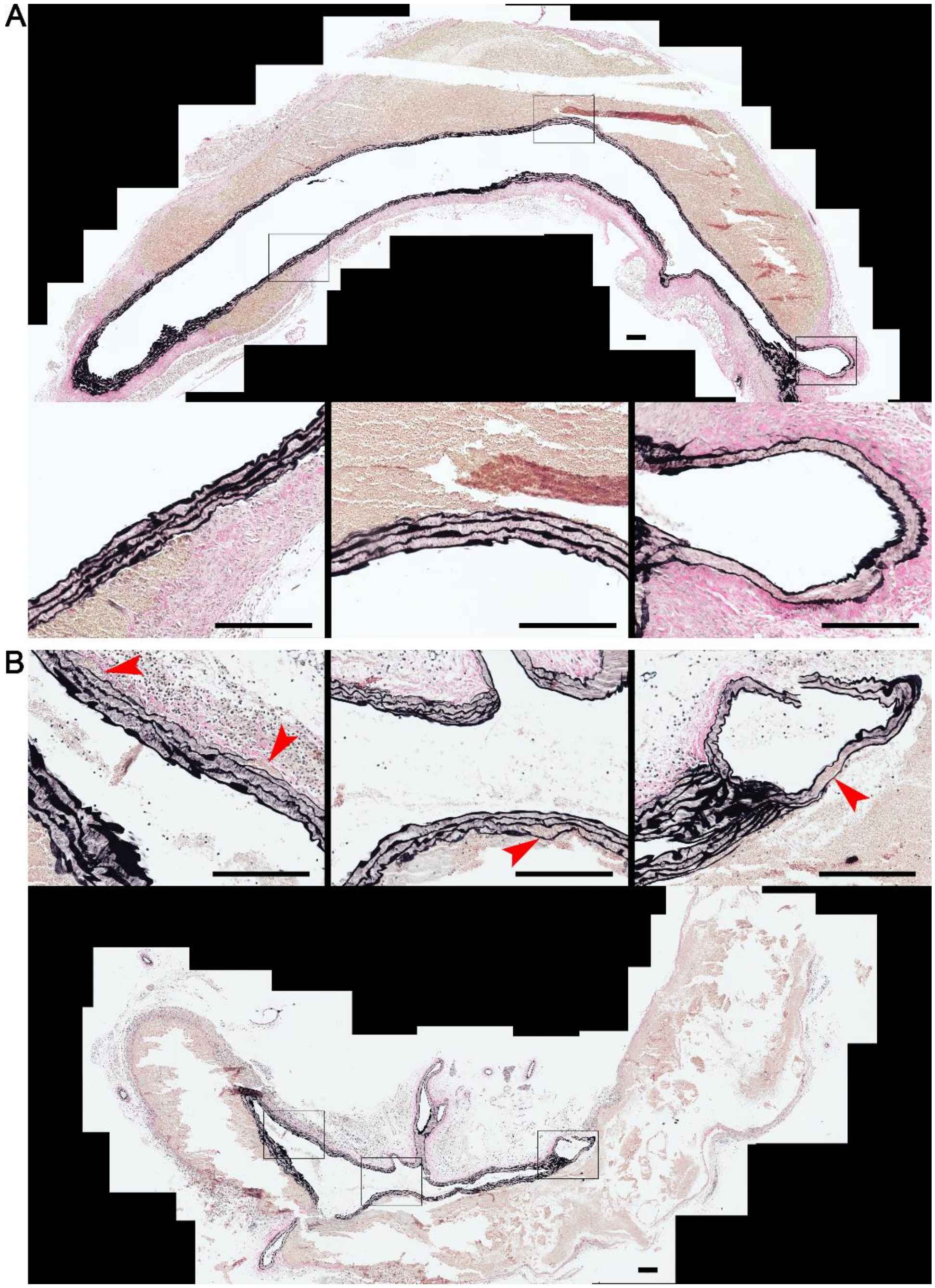

Supplemental Figure 2: AngII induces suprarenal aortic rupture and pseudoaneurysms but either no or limited medial dissection. Adult male *Apoe*$^{-/-}$ mice were infused with AngII for 7 days and longitudinal sections of the suprarenal abdominal aorta were analyzed by Verhoeff-Van Gieson stain. **(A)** Pseudoaneurysm of the aortic wall (black and pink colors) with subadventitial hematoma (brown color). Selected higher magnification views (of boxes in low magnification view) showing no blood extravasation between the elastic laminae of the aorta (left and center panels) and an artery branch (right panel). (**B**) One of 3 pseudoaneurysms sectioned longitudinally that revealed 4 areas with limited blood accumulation (red arrowheads) within the outermost lamina of the aorta (left and center panels) and within the two laminae of an artery branch (right panel). Composite photomicrographs, scale bars = 200 µm.

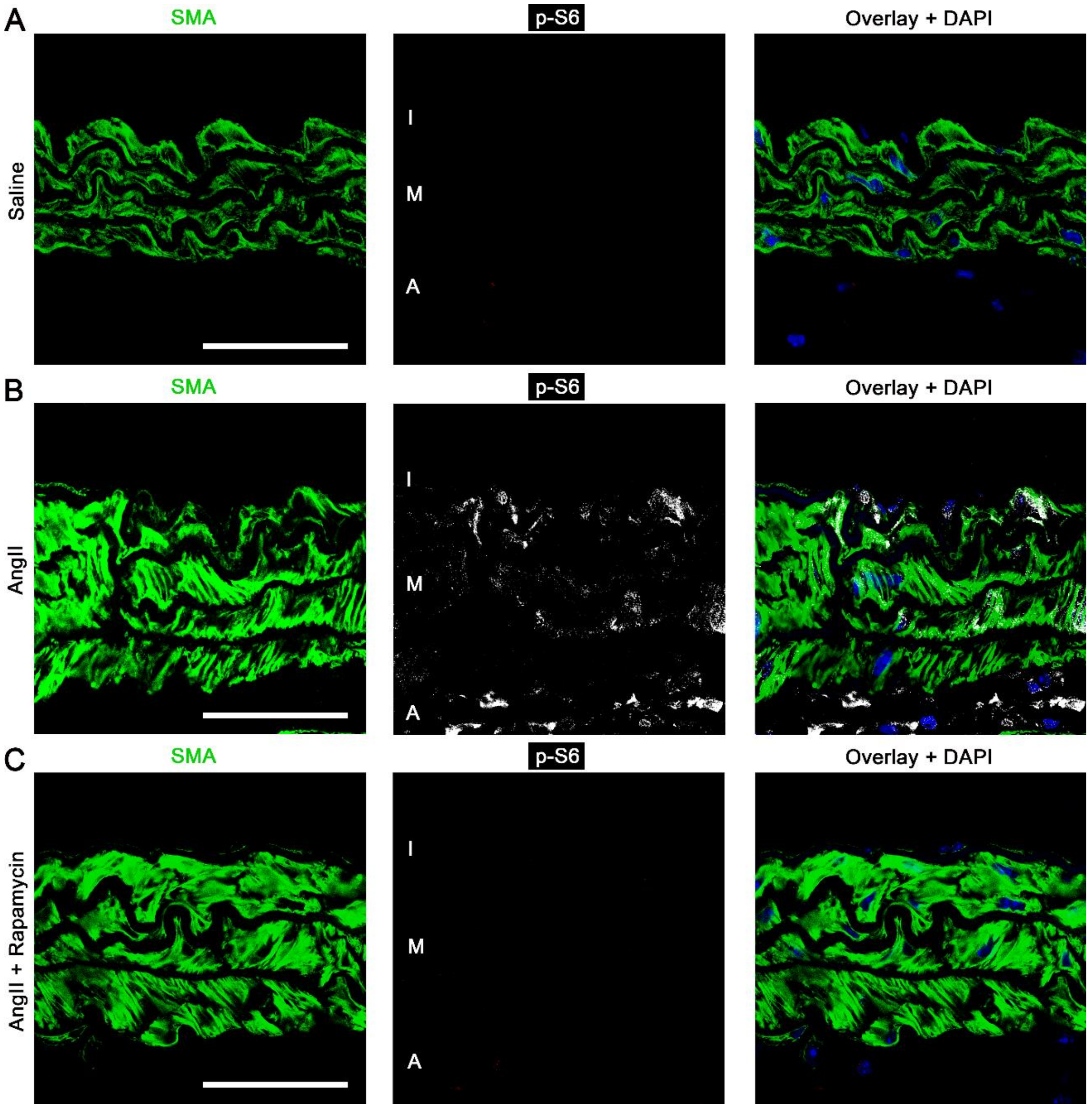

Supplemental Figure 3: AngII-induced mTOR signaling in the vascular wall was inhibited by rapamycin. Adult male *Apoe*$^{-/-}$ mice were infused with (**A**) saline, (**B**) AngII, or (**C**) AngII with rapamycin treatment for 7 days. Transverse sections of the suprarenal abdominal aorta were examined by confocal microscopy for smooth muscle α-actin (SMA, green) and phospho-S6 (white) expression in the intima (I), media (M), and adventitia (A) with overlays and DAPI-labeled nuclei (blue), scale bars = 50 μm. These images are of lower magnification (containing all 3 vascular wall layers and include saline-infused control and SMA expression to confirm identity of the media) than the Figure 5C panels.

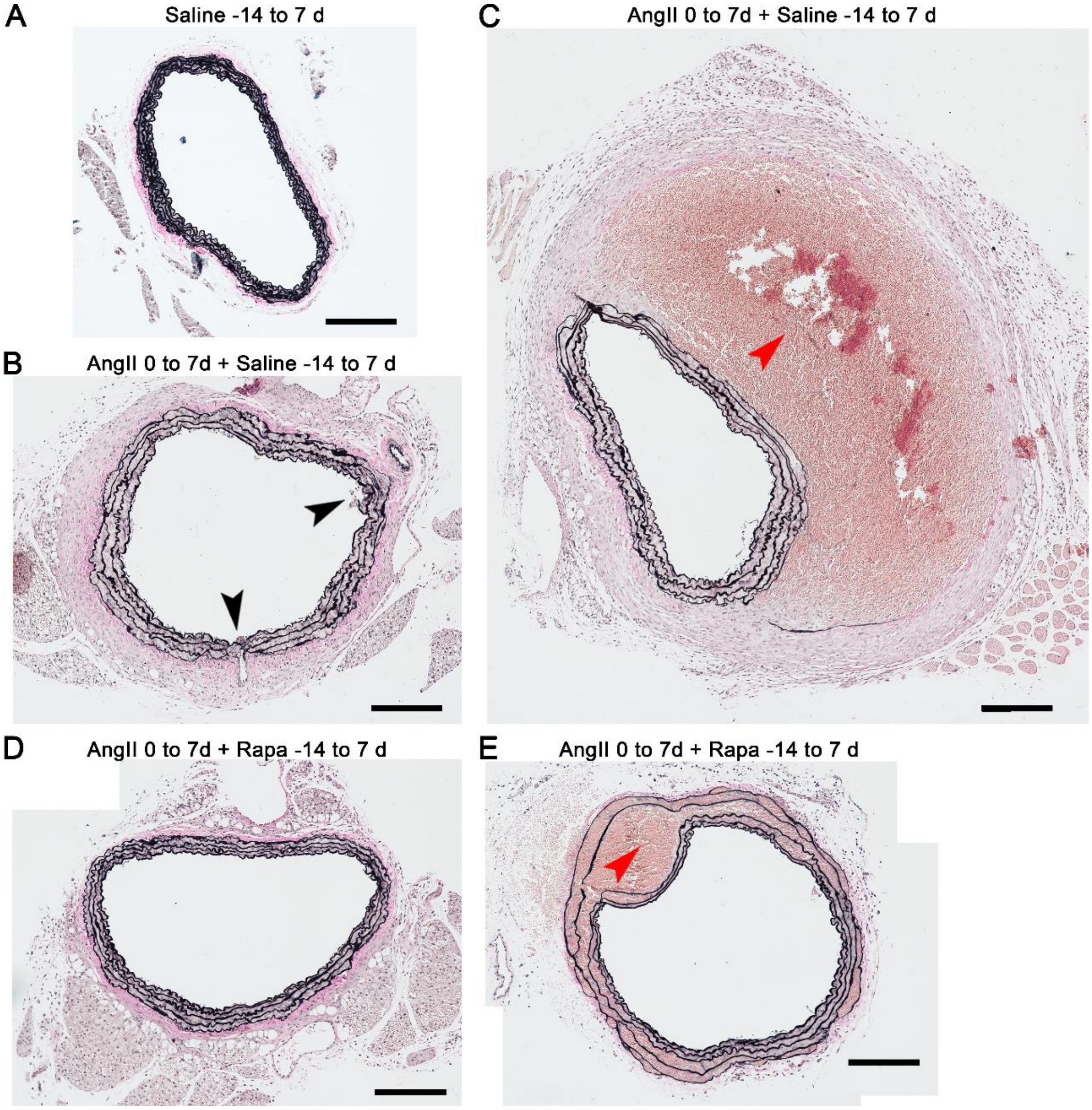

Supplemental Figure 4: Rapamycin pretreatment prevents aortic rupture and pseudo-aneurysm but exacerbates medial dissection. Adult male *Apoe*$^{-/-}$ mice were infused with AngII at 1,000 ng/kg/min s.c. for 7 days (0 to 7d) and treated with saline or rapamycin (Rapa) at 2 mg/kg/d i.p. q.d. starting 2 weeks earlier and continuing concurrent with AngII (-14 to 7d). Transverse sections of the suprarenal abdominal aorta were analyzed by Verhoeff-Van Gieson stain. (**A**) Saline-treated aorta showing closely approximated elastic laminae. (**B**) AngII-treated aorta without hematoma showing more widely spaced elastic laminae; note artifacts of irregular intima and apparent media separation at origins of branches (black arrowheads). (**C**) AngII-treated aorta with hematoma showing large pseudoaneurysm (red arrowhead). (**D**) AngII plus rapamycin-treated aorta without hematoma also showing widely spaced elastic laminae compared to saline-treated controls. (**E**) AngII plus rapamycin-treated aorta with hematoma showing extensive medial dissection with erythrocyte accumulation between the elastic laminae (red arrowhead) though sparing the innermost lamina. Composite photomicrographs, scale bars = 200 μm.

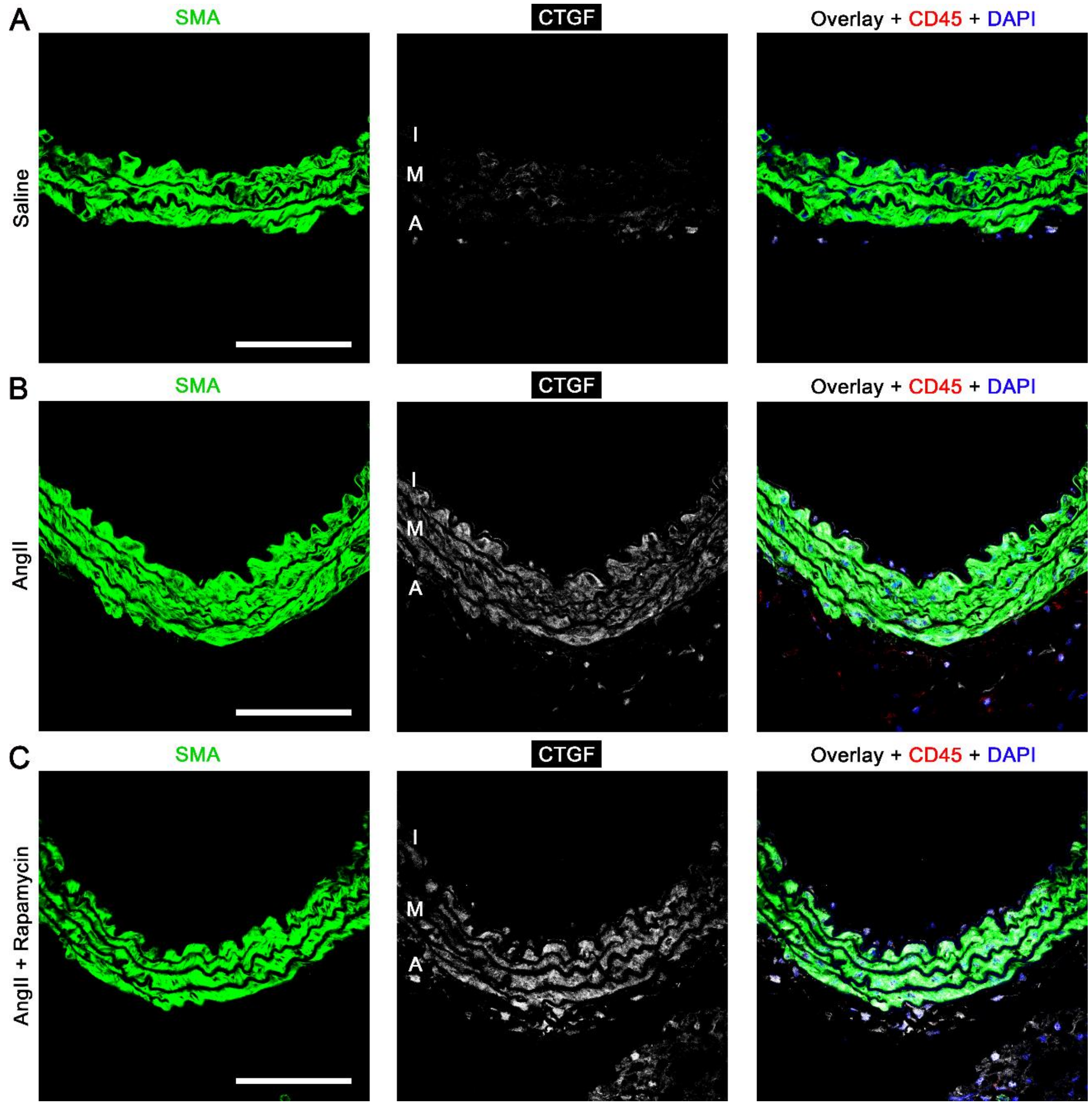

Supplemental Figure 5: AngII-induced CTGF expression in the vascular wall was not inhibited by rapamycin. Adult male *Apoe*$^{-/-}$ mice were infused with (**A**) saline, (**B**) AngII, or (**C**) AngII with rapamycin treatment for 3 days. Transverse sections of the suprarenal abdominal aorta were examined by confocal microscopy for smooth muscle α-actin (SMA, green) and CTGF (white) expression in the intima (I), media (M), and adventitia (A) with overlays including the pan-leukocyte marker CD45 (red) and DAPI-labeled nuclei (blue), scale bars = 100 μm.

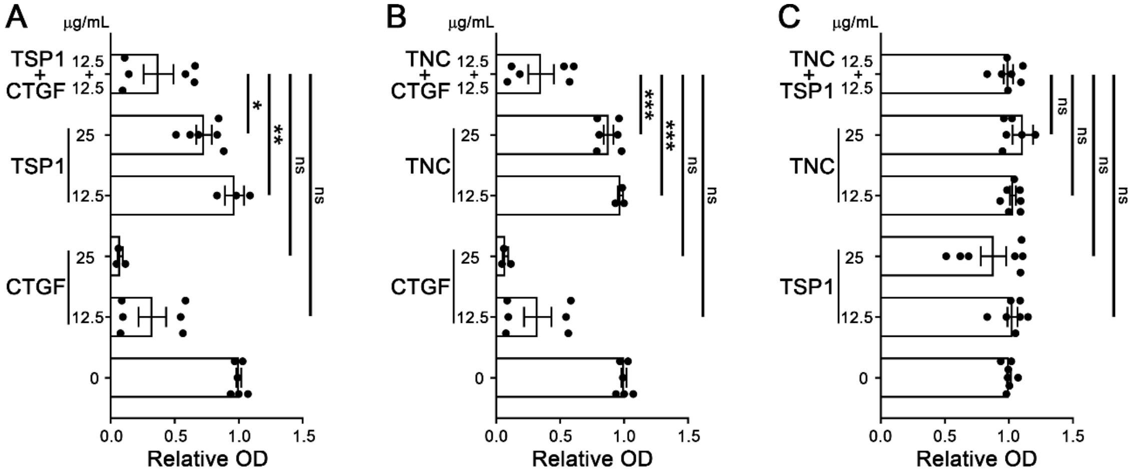

Supplemental Figure 6: No counter-adhesive interactions among matricellular proteins. Colorimetric assay for number of murine aortic SMCs adherent to fibronectin-coated plates after 1 h following cell pretreatment with (**A**) CTGF and/or thrombospondin-1 (TSP1), (**B**) CTGF and/or tenascin-C (TNC), and (**C**) thrombospondin-1 and/or tenascin-C at 12.5 and 25 µg/mL for 45 min (n = 3-7, pooled from 2 experiments); OD_{405} readings normalized to untreated controls. Individual data shown, bars represent mean ± SEM, ns not significant, *$P < 0.05$, **$P < 0.01$, ***$P < 0.001$, 1-way ANOVA with Tukey's multiple comparisons test. Additive interactions are defined as the effects from combined matricellular proteins at a particular dose (12.5 µg/mL, each) as equal to that of both individual matricellular proteins at twice that dose (25 µg/mL), and synergistic interactions are defined as the effects from combined matricellular proteins at a particular dose (12.5 µg/mL, each) as greater than that of both individual matricellular proteins at twice that dose (25 µg/mL). No positive interactions among the matricellular proteins were identified by these definitions.
